# Functional screening reveals HORMAD1-driven gene dependencies associated with translesion synthesis and replication stress tolerance

**DOI:** 10.1038/s41388-022-02369-9

**Published:** 2022-06-29

**Authors:** Dalia Tarantino, Callum Walker, Daniel Weekes, Helen Pemberton, Kathryn Davidson, Gonzalo Torga, Jessica Frankum, Ana M. Mendes-Pereira, Cynthia Prince, Riccardo Ferro, Rachel Brough, Stephen J. Pettitt, Christopher J. Lord, Anita Grigoriadis, Andrew NJ Tutt

**Affiliations:** 1 Breast Cancer Now Research Unit, King’s College London, London, UK; 2 School of Cancer and Pharmaceutical Sciences, King’s Health Partners AHSC, Faculty of Life Sciences and Medicine, King’s College London, London, UK; 3 The Breast Cancer Now Toby Robins Research Centre, The Institute of Cancer Research, London, UK; 4 The CRUK Gene Function Laboratory, The Institute of Cancer Research, London, UK

**Keywords:** Breast cancer, Genomic instability

## Abstract

HORMAD1 expression is usually restricted to germline cells, but it becomes mis-expressed in epithelial cells in ~60% of triple-negative breast cancers (TNBCs), where it is associated with elevated genomic instability (1). *HORMAD1* expression in TNBC is bimodal with HORMAD1-positive TNBC representing a biologically distinct disease group. Identification of HORMAD1-driven genetic dependencies may uncover novel therapies for this disease group. To study HORMAD1-driven genetic dependencies, we generated a SUM159 cell line model with doxycycline-inducible HORMAD1 that replicated genomic instability phenotypes seen in HORMAD1-positive TNBC (1). Using small interfering RNA screens, we identified candidate genes whose depletion selectively inhibited the cellular growth of HORMAD1-expressing cells. We validated five genes (*ATR*, *BRIP1*, *POLH*, *TDP1* and *XRCC1*), depletion of which led to reduced cellular growth or clonogenic survival in cells expressing HORMAD1. In addition to the translesion synthesis (TLS) polymerase *POLH*, we identified a HORMAD1-driven dependency upon additional TLS polymerases, namely *POLK*, *REV1*, *REV3L* and *REV7*. Our data confirms that out-of-context somatic expression of *HORMAD1* can lead to genomic instability and reveals that HORMAD1 expression induces dependencies upon replication stress tolerance pathways, such as translesion synthesis. Our data also suggest that *HORMAD1* expression could be a patient selection biomarker for agents targeting replication stress.

## Introduction

Triple negative breast cancers (TNBCs) are a relatively heterogeneous breast cancer subtype, broadly characterised by the absence of the oestrogen receptor (ER), progesterone receptor (PR) and HER2 (ERBB2), found in other subtypes of the disease [1]. Despite recent advances in the targeted treatment of TNBC (for example the use of PARP inhibitors or platinum salts in *BRCA1* or *BRCA2* mutated breast cancer [2, 3], or the use of atezolizumab in PD-L1 positive TNBC [4] for subsets of patients), targeted treatments based upon an understanding of the molecular composition of the disease are not as yet widely available. Nevertheless, there is an understanding that TNBCs, when taken as a whole, exhibit high levels of genomic instability compared to other breast cancer subtypes, suggesting a feature that could, in principle, be targeted. This genomic instability can be partly attributed to the defects in DNA repair by homologous recombination caused by *BRCA1/2* mutation [5–10] or the inactivation of other HR-associated genes [11, 12] and could induce dependencies upon permissive and potentially targetable oncogenic mutations, most likely in mechanisms associated with the DNA damage response and DNA replication stress tolerance pathways [13].

Previously, we found that HORMAD1, a protein normally only expressed in meiotic cells, is bi-modally expressed in TNBC, with 60% of tumours showing high-level expression, while the other 40% showing little to no expression [14]. In meiotic cells, HORMAD1 is involved in the generation and processing of double strand DNA breaks, as part of the pairing of homologous chromosomes and chromosomal synapsis [15]. When illegitimately expressed in human cancers, HORMAD1 expression is associated with elevated genomic instability [14, 16]. Whilst we found that HORMAD1 expression leads to impaired RAD51-dependent homologous recombination in isogenic murine embryonic stem cells and in breast cancer models, others have suggested that HORMAD1 expression enhances homologous recombination in models of other genomically unstable cancer types, such as lung adenocarcinomas [17, 18]. Despite this inconsistency, which may reflect the effects of HORMAD1 depletion on cell cycle in differing contexts, it is clear from multiple studies that HORMAD1 expression in cancer positively associates with increased genomic instability and poor prognosis [14, 16, 19]. Since HORMAD1 expression is largely restricted to malignant cells, and given its bimodal expression, HORMAD1 may be therapeutically targetable if synthetic lethal interactions i.e., genetic dependencies associated with HORMAD1 expression, can be identified. To this aim, we generated an isogenic TNBC SUM159 cell line model with doxycycline-inducible HORMAD1. HORMAD1 expression caused genomic instability, as measured by increased levels of aberrant nuclear structures (micronuclei, nuclear buds and nucleoplasmic bridges) and increased γH2AX foci formation. We then used small interfering RNA (siRNA) screening to identify genes that lead to a genetic dependency in HORMAD1-expressing cells. We validated five HORMAD1-driven gene dependencies (*ATR*, *BRIP1*, *POLH*, *TDP1* and *XRCC1*) in SUM159 and isogenic models of the non-malignant cell lines RPE1 and MCF10A. We found that, in addition to sensitivity to depletion of POLH, HORMAD1 induced a functional dependency on other TLS polymerases, namely POLK, REV1, REV3L and REV7. Our data indicate that HORMAD1 expression induces a functional dependency on replication stress tolerance pathways, such as TLS and suggests that dependency might be exploited by the development of potent and specific drug-like small molecule inhibitors of TLS.

## Results

### siRNA screening identifies candidate HORMAD1-induced gene dependencies

To identify genetic dependencies associated with illegitimate HORMAD1 expression we generated SUM159 cell lines that expressed inducible high levels of HORMAD1 when exposed to doxycycline. We selected SUM159 cells for this purpose as: (i) this cell line was derived from a TNBC and possesses a pathogenic *p53* mutation, making this relevant to the TNBC context we wished to understand; (ii) SUM159 cells lack endogenous HORMAD1 expression [14, 20]; and (iii) SUM159 cells were known to be amenable to siRNA screening [21]. To generate a controlled experimental system, we performed single cell cloning of SUM159 cells prior to and post transduction of an inducible expression construct in a pINDUCER20-HORMAD1 lentivirus [22], and selected two clones for further experiments. We confirmed doxycycline-induced expression of HORMAD1 in these clones and also showed that the HORMAD1 expression level achieved in these models is comparable to that found in the endogenous HORMAD1 expressing breast cancer line MDA-MB-436 (Fig. S1A, B). Previous work has suggested context-dependent effects of HORMAD1 on DNA damage [14, 20]. In our SUM159 clones, induction of HORMAD1 increased the proportion of nuclei with >5 γH2AX foci (Fig. S1C, D) and increased the number of aberrant nuclear structures, namely micronuclei, nuclear buds and nucleoplasmic bridges compared to control SUM159 engineered with a pINDUCER20-GFP, which allowed expression of GFP upon doxycycline induction (Fig. S1E–H), in line with our previous findings [14].

**Fig. 1:**
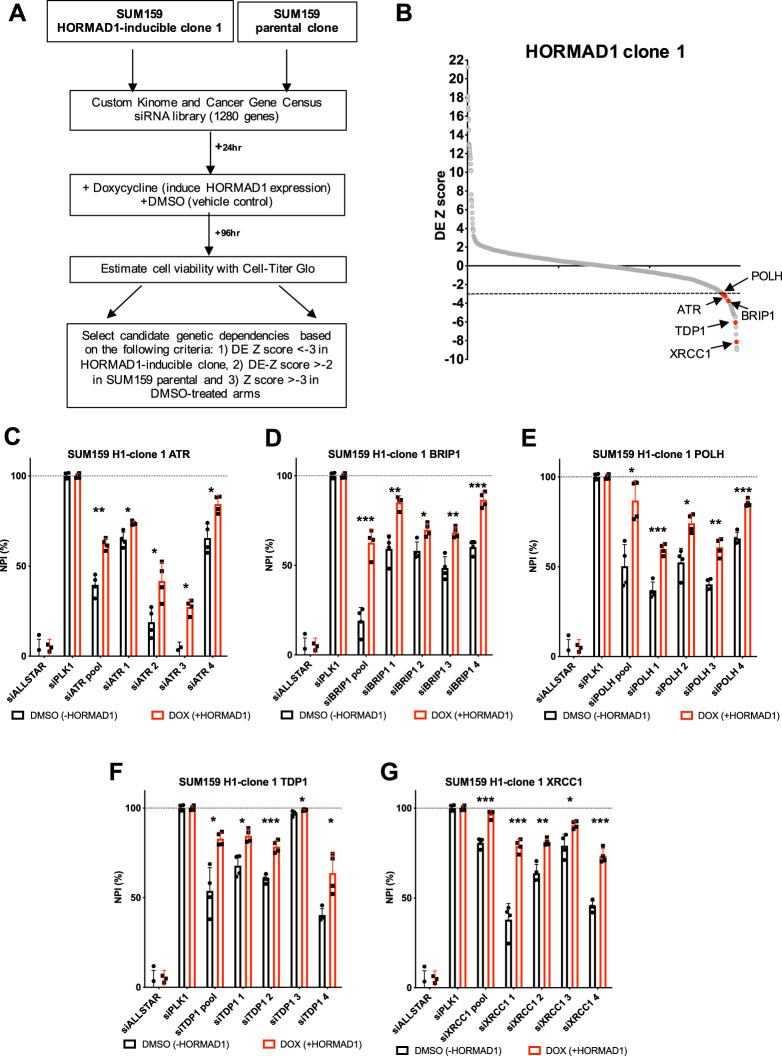
DDR-focused RNAi screen identifies HORMAD1-driven genetic dependencies. **A** Schematic diagram describing workflow for parallel siRNA screens in parental SUM159, and clonally-derived HORMAD1-inducible SUM159. Cells were reverse-transfected into siRNA-containing 384-well plates, and doxycycline added 24 h post-transfection. Cell viability was measured 5 days post-transfection using CellTiter-Glo. CellTiter-Glo readings were converted into Z scores, and doxycycline-inducible effects were identified using drug effect (DE) Z-scores. Candidate genetic dependencies were selected using the following criteria: 1) DE Z-score < -3 in HORMAD1-inducible clone 1, 2) DE Z-score > -2 in SUM159 parental clone and 3) Z-score > -3 in DMSO-treated arms. **B** Scatter plot displaying the distribution of DE-Z scores in HORMAD1-inducible SUM159 clone 1. Negative DE Z-scores are indicative of HORMAD1-driven dependencies. A numerical threshold of DE Z-score < -3 was used for candidate selection. Fourteen candidate DDR genetic dependencies were interrogated in secondary deconvolution experiments, of which 5 were validated as HORMAD1-induced genetic dependencies (marked in red). **C**–**G** Bar plots displaying increased normalised percentage inhibition (NPI) of clonally-derived HORMAD1-inducible SUM159 cells (+DOX/ + HORMAD1 vs. -DOX/-HORMAD1) transfected with an siRNA pool or four individual siRNAs targeting *ATR*, *BRIP1*, *POLH*, *TDP1* and *XRCC1* and exposed to HORMAD1 expression for 4 days. Non-targeting (siALLSTAR) and targeting (siPLK1) siRNAs were used as normalisation controls. Error bars indicate SD from mean effects (*n* = 3), *p* values represent multiple Student *t*-tests (***p = < 0.0001, **p = < 0.001, *p = < 0.05).

We then performed siRNA screening in one HORMAD1-expressing isogenic SUM159 clone (H1-clone 1), as well as in the corresponding parental SUM159 cell line (Fig. 1A). Our siRNA library targeted 1280 genes with pools of 4 siRNAs, which included 720 genes encoding the human kinome and kinase-related genes, 80 tumour suppressor genes, and 480 genes featuring in the Cancer Gene Census list [23] (Table S1). Details related to the siRNA library were published elsewhere [24]. For the screen, cells were reverse-transfected with the siRNA library in 384-well plates. Twenty-four hours after transfection replica plates were exposed either to doxycycline, to induce HORMAD1 expression, or to the doxycycline vehicle, DMSO. Cell viability was estimated five days post-transfection using CellTiter-Glo (Fig. 1A). In order to compare between different experimental arms, cell viability data were first converted into Z-scores and quality control assessments conducted as described previously [25, 26] (Fig. S2). To identify genetic dependencies induced by HORMAD1 expression, we used an analytical approach commonly used in siRNA screens to identify drug sensitisation effects [26], drug effect (DE) Z scores, which allowed the effect of each siRNA on cell viability to be compared in the presence and absence of doxycycline/HORMAD1 expression. DE-Z scores were calculated for each siRNA for both H1-clone 1 and parental SUM159 cells (Table S2). In this case, negative DE Z-scores indicated that HORMAD1 expression caused sensitivity to the siRNA. As the Z -3 threshold is roughly equivalent to three standard deviations from the median effect, we considered siRNAs with a DE-Z score < -3 in H1-clone 1 and > -2 in parental SUM159 cells as candidate HORMAD1-related genetic dependencies. As an additional filter, we removed siRNAs which, in the absence of doxycycline caused profound cell growth inhibition (Z < -3), as this suggests they target a core essential gene and cause common artefacts in such screens. Through this stringent approach, we identified 63 candidate HORMAD1-associated genetic dependencies (Table S3; Fig. 1B).

We carried out manual annotation and STRING protein network analysis [27] to evaluate which functional groups and pathways the 63 candidate HORMAD1-related genetic dependencies fall into (Table S4, 5). We found the following most highly enriched Reactome pathways [27]: (1) Transcriptional Regulation by TP53 (represented by ATR, BRIP1, CREBBP, DAXX, FANCC, NUAK1, PIP4K2B, PRKAA2, PRKAB1, PTEN, RRM2B, STK11, TOPBP1), 2) DNA repair (represented by ATR, BRIP1, DCLRE1A, ERCC4, FANCC, NTHL1, PNKP, POLH, TDP1, TOPBP1, WHSC1, XRCC1), 3) Regulation of TP53 Activity (represented by ATR, BRIP1, DAXX, NUAK1, PIP4K2B, PRKAA2, PRKAB1, STK11, TOPBP1), and 4) DNA Double-Strand Break Repair (represented by ATR, BRIP1, ERCC4, POLH, TDP1, TOPBP1, WHSC1, XRCC1). In addition, the top two enriched KEGG pathways were: 1) the Fanconi Anaemia pathway (represented by ATR, BRIP1, ERCC4, FANCC and POLH) and (2) the FoxO signalling pathway (represented by CREBBP, NLK, PRKAA2, PRKAB1, PTEN and STK11). Further details related to their functional annotations are described in Table S4, 5.

### Validation of HORMAD1-induced DNA damage response genetic dependencies

Of the 63 genes whose depletion resulted in a DE-Z < -3, 14 function in the canonical DNA damage response (DDR) (Table S4). As HORMAD1 upregulation is associated with increased genomic instability [14, 16] and expression of HORMAD1 in the SUM159 model used recapitulates previously-reported genomic instability phenotypes (Fig. 1), we have initially focused on these 14 DDR-related genes for further validation (Fig. S3). To exclude further analysis of “off-target” effects of RNAi, we performed a secondary validation screen using four individual siRNA oligonucleotides. The secondary validation screen was performed in three cell lines: the HORMAD1-inducible isogenic SUM159 clone, the parental SUM159 clonal cell line from the original screen and an additional SUM159 isogenic clone with doxycycline-inducible expression of GFP, used as a means to assess the possibility that the pInducer vector expression system and/or doxycycline exposure alone caused genetic dependencies. GFP induction in this system had not led to an increase in the number of aberrant nuclear structures, suggesting it would be an appropriate negative-control model (Fig. S1G and H). Gene effects were considered ‘on-target’ if two or more of the individual siRNAs present in the original siRNA pool resulted in significant doxycycline-induced cell inhibitory effects in the HORMAD1-expressing line. In addition, we excluded genes for which the same siRNAs resulted in doxycycline-induced cell inhibitory effects in both GFP-expressing and parental doxycycline-treated cells, as these were likely to represent sensitising effects of doxycycline or associated effects of exogenous protein expression itself. Finally, we confirmed the efficacy of each siRNA oligonucleotide and siRNA pool using RT-qPCR analysis. For the validated genes, all siRNAs resulted in at least 30% gene knockdown (Fig. S4). According to these criteria, the following genes were validated as “on-target” HORMAD1-induced genetic dependencies: *ATR*, *BRIP1*, *POLH*, *TDP1* and *XRCC1* (Fig. 1C–G; Fig. S5).

**Fig. 2:**
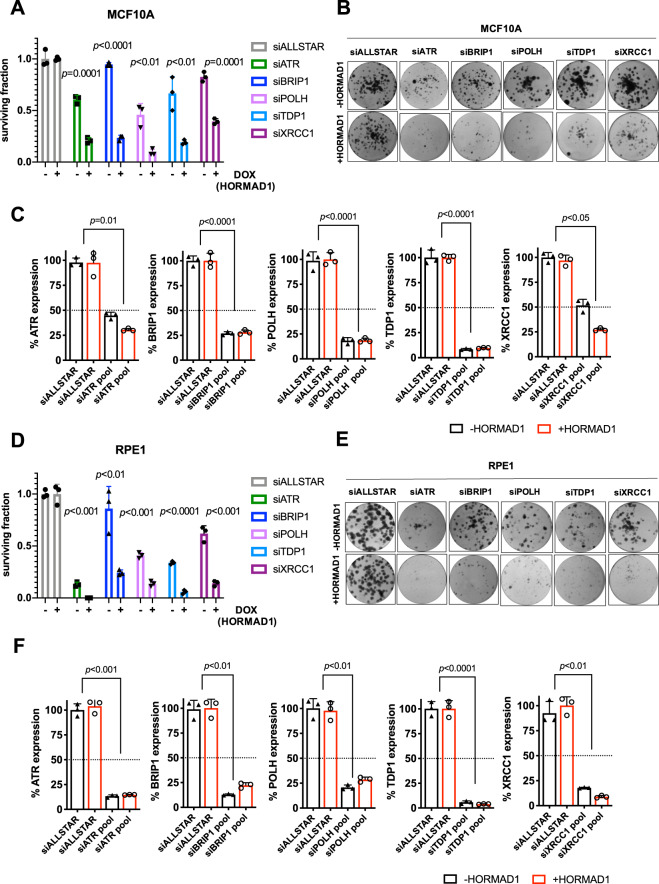
HORMAD1 drives ATR, BRIP1, POLH, TDP1 and XRCC1 dependencies in multiple cellular models. **A** Bar plot displaying reduced colony counts of MCF10A cells (+DOX/ + HORMAD1 *vs*. -DOX/-HORMAD1) transfected with an siRNA pool targeting *ATR*, *BRIP1*, *POLH, TDP1* and *XRCC1* exposed to HORMAD1 expression for 14 days (in total). Non-targeting (siALLSTAR) siRNA was used as normalisation control. Error bars indicate SD from mean effects (*n* = 3), *p*-values represent multiple Student *t*-tests. **B** Representative colony images from experiment **A**. **C** Bar plot displaying the percentage of *ATR*, *BRIP1*, *POLH, TDP1* and *XRCC1* mRNA expression following siRNA-mediated gene knockdown for experiments described in **A**, measured by RT-qPCR and normalised to *ACTB*. **D** Bar plot displaying reduced colony counts of RPE1 cells (+DOX/ + HORMAD1 *vs*. -DOX/-HORMAD1) transfected with an siRNA pool targeting *ATR*, *BRIP1*, *POLH, TDP1* and *XRCC1* and exposed to HORMAD1 expression for 14 days (in total). Non-targeting (siALLSTAR) siRNA was used as normalisation control. Error bars indicate SD from mean effects (*n* = 3), *p*-values represent multiple Student *t*-tests. **E** Representative colony images from experiment **D. F** Bar plot displaying the percentage of *ATR*, *BRIP1*, *POLH, TDP1*, and *XRCC1* mRNA expression following siRNA-mediated gene knockdown for experiments described in **D**, measured by RT-qPCR and normalised to *ACTB*.

Next, we investigated whether these genetic dependencies were specific or “private” to the genetic background of SUM159 cells, or whether they represented more penetrant [28] HORMAD1-driven dependencies. For this, we used isogenic doxycycline-inducible HA tagged-HORMAD1 expressing models of the non-transformed cell lines MCF10A and RPE1 (Fig. S6A, B, E, F). In these lines, expression levels of HA tagged HORMAD1 were comparable to those seen in the HORMAD1 positive breast cancer cell line MDA-MB-436. Interestingly, time-lapse microscopy of these cells revealed that HORMAD1 impaired cellular growth (Fig. S6C, D), which is consistent with the observation that HORMAD1 expression in somatic cells drives induction of DNA damage with consequent genomic instability. Using clonogenic survival assays, we observed significant and HORMAD1-specific reduction in single-cell colony-formation capacity, exacerbated by ATR, BRIP1, POLH, TDP1 and XRCC1 depletion in both systems (Fig. 2A–F). Taken together with our previous observations, our validation experiments suggested that *ATR, BRIP1*, *POLH*, *TDP1* and *XRCC1* genetic dependencies operated in multiple model systems and exclude conclusion that effects in our screen are private to a context specific to the SUM159 model.

Having identified HORMAD1 induced dependencies in isogenic cell line models we next performed siRNA mediated knockdown experiments in the HORMAD1 positive cell lines MDA-MB-436, HCC38, BT549 and HCC1143 for ATR, BRIP, POLH, TDP1, XRCC1 (Fig. S7). We found that only POLH knockdown led to > 50% cell inhibition in all four cells lines (Fig. S7G). Additionally, both ATR and TDP1 knockdown led to > 50% cell inhibition in three of the four cells lines tested (Fig. S7A and C). This data supports the idea that POLH, ATR and TDP1 represent penetrant sensitivities for HORMAD1 expressing cells.

Finally, ATR kinase dependency was interrogated using small molecule inhibitors of ATR kinase function (ATRi), namely VE-821, VX-970/M6620 (Merck KGaA), AZ20 and AZD6738 (AstraZeneca), two of which are currently in phase I and phase II clinical trials [29]. In clonogenic survival assays, exposure of isogenic inducible-HORMAD1 SUM159 cells to VE-821, VX-970/M6620 (berzosertib), AZ20 and AZD6738 (ceralasertib) did not reduce colony-formation capacity in a HORMAD1-dependent manner (Fig. S8A–D). Similar results were observed following treatment of isogenic inducible-HORMAD1 MCF10A and RPE1 cells with AZD6738 (Fig. S8E, F). Although there may be differences between effects of ATR inhibition and depletion [30] this reduced confidence in HORMAD1 induced ATR dependency. Given the interest in translesion synthesis (TLS) polymerases as therapeutic targets in cancer [31–33] and our observation that all the HORMAD1 expressing breast cancer cell lines showed sensitivity to POLH knockdown (Fig. S7), we further investigated how the silencing of *POLH* and a wider group of TLS polymerases, affected the viability of HORMAD1-expressing cells.

### Orthogonal validation of POLH as an HORMAD1-induced genetic dependency

**Fig. 3:**
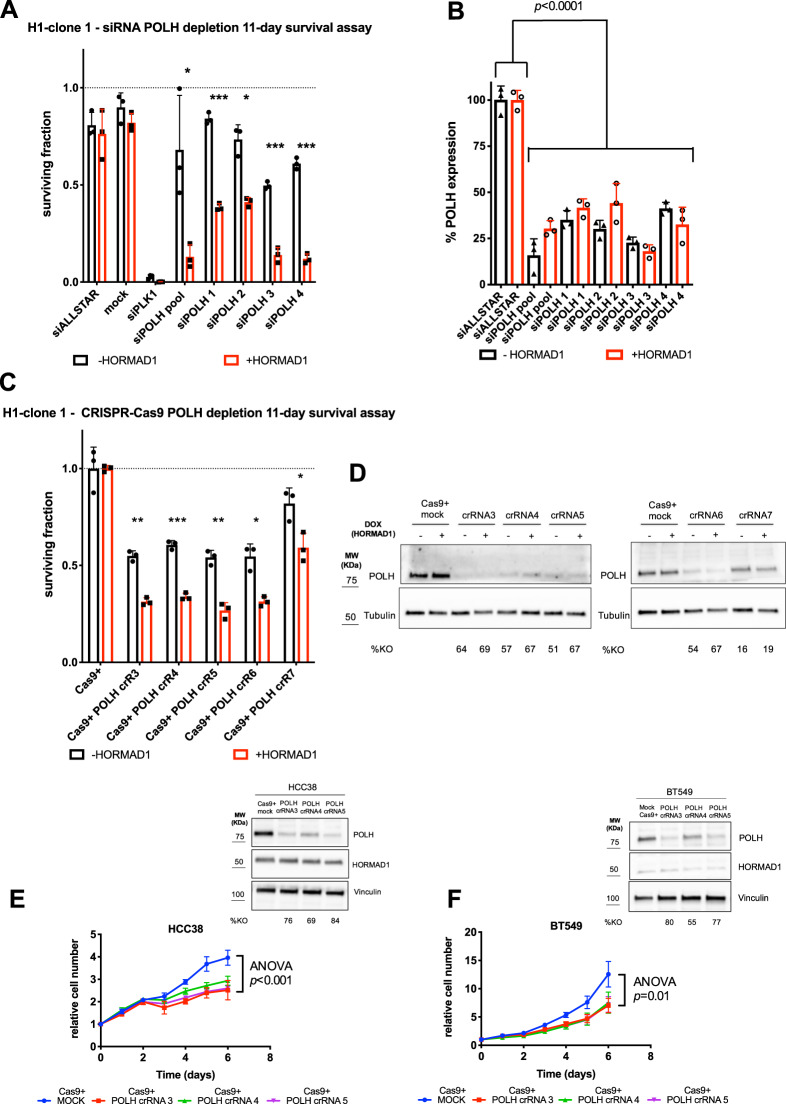
Additional validation of HORMAD1-driven POLH dependency. **A** Bar plot displaying reduced surviving fractions of clonally-derived HORMAD1-inducible SUM159 cells (+DOX/ + HORMAD1 *vs*. -DOX/-HORMAD1) transfected with an siRNA pool or 4 individual siRNAs targeting *POLH* and exposed to HORMAD1 expression for 14 days (in total). Non-targeting (siALLSTAR) and targeting (siPLK1) siRNAs were used as transfection controls and surviving fractions calculated from mock-transfected cells. Error bars indicate SD from mean effects (*n* = 3), *p*-values represent multiple Student t tests (***p = < 0.0001, **p = < 0.001, *p = < 0.05). **B** Bar plot displaying the percentage of *POLH* mRNA expression following siRNA-mediated depletion of *POLH* described in **A**, measured by RT-qPCR and normalised to *ACTB*. **C** Bar plot displaying reduced surviving fractions of clonally-derived HORMAD1-inducible SUM159 cells (+DOX/ + HORMAD1 *vs*. -DOX/-HORMAD1) expressing constitutive Cas9-mCherry, transfected with 5 Edit-R crRNAs targeting *POLH*, and exposed to HORMAD1 expression for 14 days (in total). Surviving fractions were calculated relative to Cas9-expressing mock-transfected controls. Error bars indicate SD from mean effects (*n* = 3), *p*-values represent multiple Student *t*-tests (***p = < 0.0001, **p = < 0.001, *p = < 0.05). **D** Western blot analysis of POLH protein knockout from experiment **C**. **E**, **F** *Left*, growth curves displaying reduced cellular growth of HORMAD1-expressing breast cancer cell lines **E** HCC38 and **F** BT549 expressing constitutive Cas9-mCherry and bulk-transfected with 3 *POLH*-targeting Edit-R crRNAs. Cell number was normalised relative to T0 counts. Error bars indicate SD from mean effects (*n* = 3). *p*-values represent two-way repeated-measures ANOVA. *Right*, western blot analysis of HORMAD1 expression and POLH protein knockout from experiments described in *left* panel.

As our screen had been conducted in the context of an acute 5-day exposure to HORMAD1 we wished to assess whether dependency upon POLH occurred in SUM159 cells adapted to expressing HORMAD1 over a longer time period. Both longer-term expression of HORMAD1 (14 days in total) and continuous HORMAD1 expression for 21.5 weeks resulted in a significant decrease in cellular viability following siRNA-mediated depletion of *POLH*, confirmed by RT-qPCR (Fig. 3A, B; Fig. S9A, B). Given the potential off-target effects of siRNA transfections, we sought to validate on-target *POLH* sensitivity using the orthogonal technique of Edit-R CRISPR-Cas9 mediated gene editing to deplete the wild-type *POLH* gene product. The effect of HORMAD1 on cellular sensitivity to *POLH* depletion was confirmed 11 days after guide transfection (Fig. 3C, D). Finally, we investigated whether *POLH* depletion would inhibit cellular growth in two TNBC cell lines expressing endogenous HORMAD1, namely HCC38 and BT549. By tracking cell population growth with Incucyte microscopy, we found that both models displayed reduced cellular growth following *POLH* editing (Fig. 3E, F), despite the limitations of variable Edit-R guide and CRISPR-Cas9 transfection efficiency and consequent incomplete gene editing within a bulk transfected population. Taken together, our data demonstrate that HORMAD1 expression leads to a dependency on the TLS polymerase POLH that is not private to the SUM159 model system in which it was first discovered.

### HORMAD1 expression leads to a functional dependency on multiple translesion synthesis proteins

**Fig. 4:**
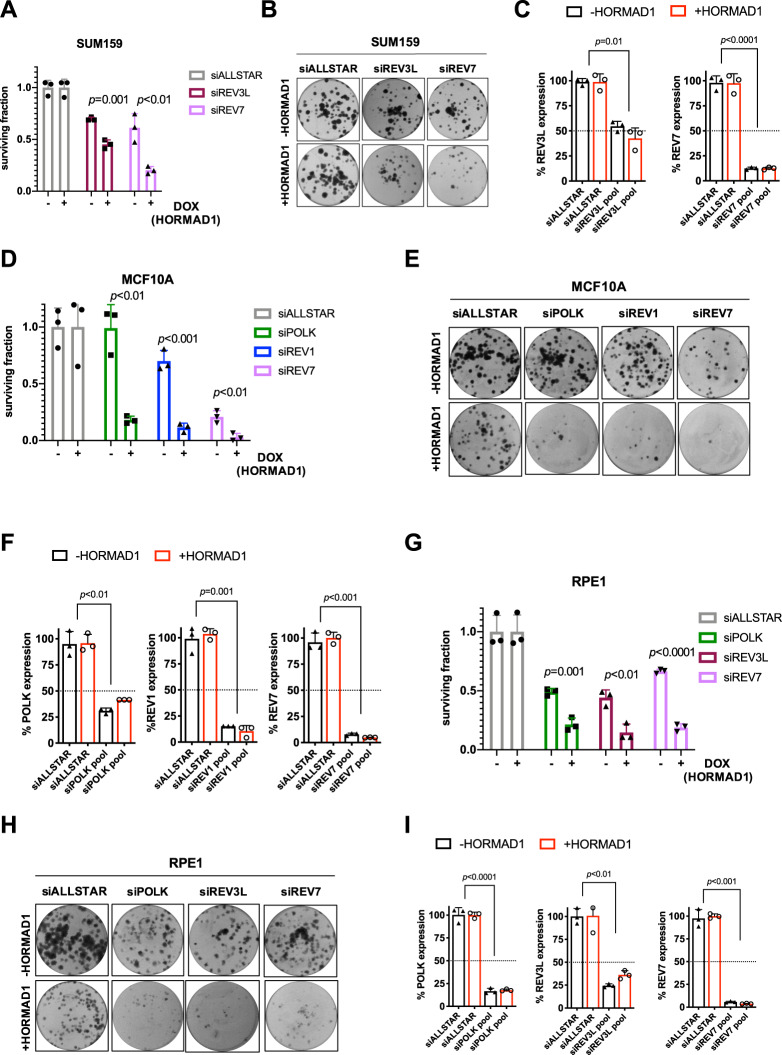
HORMAD1 drives broad genetic dependency on TLS polymerases. **A** Bar plot displaying reduced colony counts of SUM159 cells (+DOX/ + HORMAD1 *vs*. -DOX/-HORMAD1) transfected with an siRNA pool targeting *REV3L* and *REV7* and exposed to HORMAD1 expression for 14 days (in total). Non-targeting (siALLSTAR) siRNA was used as normalisation control. Error bars indicate SD from mean effects (*n* = 3), *p*-values represent multiple Student *t*-tests. **B** Representative colony images from experiment **A. C** Bar plot displaying the percentage of *REV3L* and *REV7* mRNA expression following siRNA-mediated gene knockdown for experiments described in **A**, measured by RT-qPCR and normalised to *ACTB*. **D** Bar plot displaying reduced colony counts of MCF10A cells (+DOX/ + HORMAD1 *vs*. -DOX/-HORMAD1) transfected with an siRNA SMARTpool targeting *POLK*, *REV1* and *REV7* and exposed to HORMAD1 expression for 14 days (in total). Non-targeting (siALLSTAR) siRNA was used as normalisation control. Error bars indicate SD from mean effects (*n* = 3), *p*-values represent multiple Student *t*-tests. **E** Representative colony images from experiment **D. F** Bar plot displaying the percentage of *POLK*, *REV1* and *REV7* mRNA expression following siRNA-mediated gene knockdown for experiments described in **D**, measured by RT-qPCR and normalised to *ACTB*. **G** Bar plot displaying reduced colony counts of RPE1 cells (+DOX/ + HORMAD1 *vs*. -DOX/-HORMAD1) transfected with an siRNA SMARTpool targeting *POLK*, *REV3L* and *REV7* and exposed to HORMAD1 expression for 14 days (in total). Non-targeting (siALLSTAR) siRNA was used as normalisation control. Error bars indicate SD from mean effects (*n* = 3), *p*-values represent multiple Student *t*-tests. **H** Representative colony images from experiment **G. I** Bar plot displaying the percentage of *POLK*, *REV3L,* and *REV7* mRNA expression following siRNA-mediated gene knockdown for experiments described in **G**, measured by RT-qPCR and normalised to *ACTB*.

POLH is a TLS polymerase that facilitates replication across replication-blocking DNA lesions [34]. As a wider group of TLS polymerases are involved in similar functions, we hypothesised that the observed HORMAD1-driven *POLH* dependency could extend to additional TLS polymerases. To test this, we depleted *POLI*, *POLK*, *REV1*, *REV3L* and *REV7* using siRNA and used clonogenic survival assays to test effects on clonogenic capacity following inducible HORMAD1 expression in SUM159, MCF10A and RPE1. These experiments revealed that *REV7* depletion impaired clonogenic survival to a greater extent in HORMAD1-expressing SUM159 (Fig. 4A–C), MCF10A (Fig. 4D–F) and RPE1 (Fig. 4G–I) cells. In contrast, we observed a HORMAD1-driven sensitivity to *REV3L* in SUM159 (Fig. 4A–C) and RPE1 (Fig. 4G–I) but not in MCF10A (Fig. 4D–F). We also observed a HORMAD1-driven sensitivity to *POLK* in MCF10A (Fig. 4D–F) and RPE1 (Fig. 4G–I) only, and to *REV1* in MCF10A only (Fig. 4D–F). The apparent lack of dependency on *REV3L* in MCF10A (Fig. S10D–F) and on *REV1* in SUM159 (Fig. S10A–C) and RPE1 (Fig. S10G–I) could reflect less efficient siRNA-mediated knockdown of these genes in these specific models. However, the lack of consistency across models may also reflect differences in model-specific background biological context, leading to model-enriched dependencies upon specific TLS polymerases within the family as a whole. We next performed siRNA mediated knockdown experiments in the HORMAD1 positive cell lines MDA-MB-436, HCC38, BT549 and HCC1143 for POLK, REV1, REV3L and REV7 (Fig. S11). We found that REV7 produced cell inhibition of >50% in all four lines tested (Fig. S11F). POLK knockdown produced cell inhibition of >50% in three out of four lines tested (Fig. S11A). This data supports the idea that TLS polymerases represent penetrant sensitivities in HORMAD1 expressing cells.

Taken together, our results reveal a number of genes that are essential for cellular viability following out-of-context expression of HORMAD1 and suggest that TLS may enable replication stress tolerance in cells expressing HORMAD1.

## Discussion

HORMAD1 is a meiotic gene that becomes aberrantly expressed in cancers. In this study, we developed a doxycycline-inducible HORMAD1 expression system that can be used to model the effects of HORMAD1 in mitotic cells. In line with previous publications [14, 16, 19], we found that HORMAD1 induction caused genomic instability. Consistent with this effect of out of context expression of HORMAD1, we found that tumour cells expressing HORMAD1 have specific vulnerabilities related to their ability to repair DNA damage or replicate through damaged DNA. We identified dependency upon *ATR*, *BRIP1*, *POLH*, *TDP1* and *XRCC1* as specific vulnerabilities induced by HORMAD1 expression in the TNBC SUM159 cell line model, as well as in isogenic models of the non-malignant cell lines MCF10A and RPE1.

Translesion synthesis (TLS) is a DNA damage tolerance pathway that allows cells to replicate DNA across DNA lesions, but has the potentially mutagenic effect of utilising low-fidelity DNA polymerases [34]. Mammalian cells possess at least five TLS polymerases (Pol ζ [REV3L/REV7], REV1, POLH, POLK and POLI), each of which has different, but overlapping, substrate specificities (reviewed in [35]). In addition to their role in translesion bypass, TLS polymerases mediate replication fork restart in response to hydroxyurea-induced replication fork arrest [36]. Importantly, TLS inhibition has been shown to modulate the therapeutic response to chemotherapy [31–33] and to the BRAF inhibitor Vemurafenib, in cells experiencing BRAF^V600E^ oncogene-depletion induced stress [37]. Identification of *POLH* in our primary screen, and interest in the drug discovery field in targeting translesion synthesis, led us to seek a HORMAD1-induced dependency on other TLS polymerases. In addition to *POLH*, we found that HORMAD1 expression induced a dependency on *REV7* in SUM159, MCF10A and RPE1 cell line models. We also observed a dependency on *REV3L* in SUM159 and RPE1 cells, on *POLK* in MCF10A and RPE1 cells, and on *REV1* in MCF10A cells, each of which may be more private to the genetic background of each respective cell line.

By identifying bimodal and tumour cell specific somatic expression of the meiotic protein HORMAD1 as a potential patient selection biomarker our study contributes to a growing body of evidence that TLS dependency is a tractable therapeutic target in cancer. Small molecule tool box inhibitors targeting Pol ζ, POLH and POLK have recently been described [32, 33, 38]. If potent and specific drug-like inhibitors of TLS polymerases can be further developed they may represent a novel therapeutic strategy for a majority subgroup of TNBCs and potentially other tumour sites with clearly identifiable HORMAD1 expression. A number of small-molecule TDP1 inhibitors have also been developed [39, 40] suggesting that our identification of TDP1 dependency could also be therapeutically relevant in HORMAD1-positive TNBC.

In conclusion, our data identifies a number of HORMAD1-induced genetic dependencies, which might be selectively targeted with small molecules in a group of high unmet need malignancies with readily identifiable tumour restricted expression of the meiotic protein HORMAD1.

## Materials and methods

All materials and methods are provided in the Supplementary Material.

## Supplementary information

Supplementary Material

Supplementary Table 1

Supplementary Table 2

Supplementary Table 3

Supplementary Table 4

Supplementary Table 5

Supplementary Table 6

Supplementary Table 7
