# Supplementary material for: Functional screening reveals HORMAD1-driven gene dependencies associated with translesion synthesis and replication stress tolerance
Source: Oncogene. 2022 Jun 29;41(32):3969–77. doi: 10.1038/s41388-022-02369-9 (PMC9355871; doi:10.1038/s41388-022-02369-9)
Supplement: Supplementary file 1 — Supplementary Material [file 41388_2022_2369_MOESM1_ESM.pdf]

Supplementary Material

1. Materials and Methods

2. Supplementary Figure Legends

3. Seventeen Supplementary Figures

Fig. S1: Doxycycline-inducible HORMAD1 expression drives genomic instability.
Fig. S2: RNAi screen quality control data.
Fig. S3: Fourteen DDR-associated gene candidates identified in the primary screen.
Fig. S4: Validation of siRNA gene silencing effects by RT-qPCR analysis.
Fig. S5: Deconvolution RNAi validation of 14 gene candidates.
Fig. S6: Generation of clonally-derived HORMAD1-inducible MCF10A and RPE1 cell lines.
Fig. S7: HORMAD1-associated genetic dependencies in HORMAD1 positive cell lines
Fig. S8: HORMAD1 expression does not sensitise cells to ATR inhibitors.
Fig. S9: Validation of HORMAD1-driven POLH dependency in 21.5-weeks HORMAD1-expressing SUM159 cells.
Fig. S10: Candidate hits lacking HORMAD1-driven selectivity in SUM159, MCF10A and RPE1 cells.
Fig. S11: HORMAD1-associated TLS genetic dependencies in HORMAD1 positive cell lines.
Fig. S12 – Fig. S17: Uncropped scans of western blots.

4.Supplementary References

Materials and Methods

Cell lines

The following breast cancer cell lines BT549, HCC38, HCC1143, MDA-MB-436; the immortalised mammary epithelial cell line MCF10A and the immortalised retinal pigment epithelial cell line RPE-1 were obtained from the American Type Culture Collection (ATCC, Manassas, VA, USA). The breast cancer cell line SUM159 was obtained from Asterand (Royston, UK). All cell lines were grown in 5% CO_2 at 37°C.

BT549 was grown in DMEM Medium (Gibco, ThermoFisher Scientific, Waltham, MA, USA) supplemented with 10% fetal bovine serum (FBS) (Gibco, ThermoFisher Scientific, Waltham, MA, USA) and Recombinant Human Insulin (10µg /mL; Sigma-Aldrich, St. Louis, MO, USA). HCC38 and HCC1143 were grown in RPMI-1640 Medium (Gibco, ThermoFisher Scientific, Waltham, MA, USA) supplemented with 10% fetal bovine serum (FBS) (Gibco, ThermoFisher Scientific, Waltham, MA, USA).

MDA-MB-436 was grown in RPMI-1640 Medium (Gibco, ThermoFisher Scientific, Waltham, MA, USA) supplemented with 10% fetal bovine serum (FBS) (Gibco, ThermoFisher Scientific, Waltham, MA, USA) and Recombinant Human Insulin (10µg /mL; Sigma-Aldrich, St. Louis, MO, USA).

MCF10A was grown in DMEM/F-12 (Gibco, ThermoFisher Scientific, Waltham, MA, USA) supplemented with 5% Horse Serum (Gibco, ThermoFisher Scientific, Waltham, MA, USA), EGF (20ng/mL; Peprotech, London, UK), Hydrocortisone (0.5mg/mL; Sigma-Aldrich, St. Louis, MO, USA), Cholera Toxin (100ng/mL; Sigma-Aldrich, St. Louis, MO, USA), and Recombinant Human Insulin (0.1mg/mL; Sigma-Aldrich, St. Louis, MO, USA). SUM159 cells were grown in Ham's F-12 Nutrient Mixture (Gibco, ThermoFisher Scientific, Waltham, MA, USA) supplemented with 5% FBS (Gibco, ThermoFisher Scientific, Waltham, MA, USA), HEPES (10nM; Sigma-Aldrich, St.

Louis, MO, USA), Hydrocortisone (1μg/mL; Sigma-Aldrich, St. Louis, MO, USA), and Recombinant Human Insulin (5μg/mL; Sigma-Aldrich, St. Louis, MO, USA). Each media was prepared according to the supplier's recommendations. All cell lines were grown in the presence of Penicillin-Streptomycin (Gibco, Thermo Fisher Scientific, Waltham, MA, USA) at a final concentration of 100 U/mL. Doxycycline hyclate (Sigma-Aldrich, St. Louis, MO, USA) was added to cells at a final concentration of 0.125mg/mL, 0.25mg/mL and 0.5mg/mL for SUM159, RPE1 and MCF10A cells, respectively. STR valiadated cell line stocks were used and all cell lines used were within 30 passages of this STR typing. All cells were routinely tested for mycoplasma

Lentiviral p20 HORMAD1 and GFP construct generation

HORMAD1 or GFP cDNAs were cloned into the pInducer20 lentivirus expression vector (a gift from Stephen Elledge, Addgene plasmid # 44012) according to a protocol described previously (1). Lentiviral particles were produced in HEK293T cells by co-transfection of the relevant pINDUCER20 plasmids with the pMD2.G (Addgene plasmid # 12259) and psPAX2 (Addgene plasmid # 12260) packaging vectors, which were a gift from Didier Trono. Virus-containing cell culture media was collected for downstream transduction of cell lines and the virus titre was estimated in SUM159 using serial dilution of viral supernatants and selection with 0.5mg/ml Geneticin (Gibco, Thermo Fisher Scientific, Waltham, MA, USA).

Generation of isogenic and clonal cell systems

Parental (heterogenous) SUM159 cells were isolated in 96 well plates using serial dilutions to obtain a final density of 1 cell/well. Colony formation was monitored by light microscopy and single-cell colonies were expanded to generate "parental clones". Parental clones were plated into 6-well plates, infected with lentiviral vectors at a multiplicity of infection (MOI) of 1. After two days, selection was initiated by adding 0.5

mg/mL of Geneticin (Gibco, Thermo Fisher Scientific, Waltham, MA, USA) and continued for at least three days. Single-cell clones were derived for a second time in order to achieve isogenic clonally-derived cells, with doxycycline-inducible HORMAD1 expression. Clones with doxycycline-inducible HORMAD1 were selected using western blotting, whilst GFP-expressing clones were selected using a flow cytometry. The same process was performed to obtain MCF10A and RPE1 cell lines, but without the initial selection of single cell clones.

Primary RNA Interference Screening

Cells from a SUM159 parental clone and a clonally-derived HORMAD1-expressing SUM159 sub-line, were reverse transfected with a Dharmacon SMARTpool siRNA library (Dharmacon, Lafayette, CO, USA) targeting a total of 1280 genes. This included: 720 genes encoding the kinome and kinase-related genes, 80 tumour suppressor genes, and 480 genes featured in the Cancer Gene Census (Table S1), as previously described (2). siRNA oligos were arrayed in 384-well plates which included the positive control (siPLK1; Dharmacon, Lafayette, CO, USA) and multiple non-targeting controls (siCON1 and siCON2; Dharmacon, Lafayette, CO, USA and AllStar; QIAGEN, Hilden, Germany). 20nM of siRNA and 0.05μL of transfection reagent RNAiMAX (Invitrogen, Thermo Fisher Scientific, Waltham, MA, USA) were used per well. Screens were carried out in triplicate. Twenty-four hours post-transfection, cells were treated with doxycycline (0.5 μg/ml) for induction of HORMAD1 expression, or vehicle (DMSO; Sigma-Aldrich, St. Louis, MO, USA). Cell viability was measured 5 days post-transfection using CellTiter-Glo (Promega, Madison, WI, USA). Data processing and quality control analysis were conducted using the cellHTS2 R package as described previously (3, 4). Drug Effect was calculated as follows: $\boldsymbol{x}_{\boldsymbol{DE}} = (\boldsymbol{x}_{\boldsymbol{doxycycline}} - \boldsymbol{x}_{\boldsymbol{DMSO}})$. Where: $\boldsymbol{x}_{\boldsymbol{DE}}$ = drug effect for siRNA x, $\boldsymbol{x}_{\boldsymbol{doxycycline}}$ = $\log_2$ plate-

centred luminescence value for siRNA x in the doxycycline arm, $\boldsymbol{x_{DMSO}}$ = log2 plate-centred luminescence value for siRNA x in the DMSO arm.

(DE)-Z scores were then calculated using the formula: $\boldsymbol{DE\ Z-score} = \frac{x_{DE}-\mu_{DE}}{\sigma_{DE}}$.

Where: $\boldsymbol{x_{DE}}$ = Drug Effect for siRNA x, $\boldsymbol{\mu_{DE}}$ = Median Drug Effect for entire library, $\boldsymbol{\sigma_{DE}}$ =Median Absolute Deviation of Drug Effects across entire library. This methodology was described previously (5). For subsequent interrogation, we selected genes with a DE-Z score of less than -3, indicating a HORMAD1/doxycycline induced gene sensitivity. We removed genes which also had a DE-Z of less than -2 in the parental cell line. We also removed inherently toxic genes (Z score of less than -3).

Single oligo siRNA deconvolution RNA interference validation

Secondary validation experiments were performed with the same methodology as the primary screen but using sets of four individual targeting siRNAs (Dharmacon, Lafayette, CO, USA), as well as siRNA SMARTpools (Dharmacon, Lafayette, CO, USA) utilised in the primary screen. Data were analysed and presented using the normalised percentage inhibition, NPI= $\frac{x-c_{-}}{c_{+}-c_{-}}$ *100. Where: x= raw luminescence value for siRNA x, c_{-}= average raw luminescence value for non-targeting siRNA control (siALLSTAR), c_{+}= average raw luminescence value for targeting siRNA control (siPLK1), as described previously (6). Data from triplicate wells of a single replicate are shown.

Validation of gene knockdown by RT-qPCR

Gene knockdown following treatment with each siRNA was determined by RT-qPCR. Briefly, total RNA was isolated using the RNeasy Mini kit (QIAGEN, Hilden, Germany) for 6-well plate transfections, and the MagMAX-96 Total RNA Isolation Kit (Ambion, Thermo Fisher Scientific, Waltham, MA, USA) for 96-well plate transfections and cDNA generated with the High-Capacity cDNA Reverse Transcription kit (Applied

Biosystems, Foster City, CA, USA). Reverse-transcription quantitative PCR (RT-qPCR) was performed using PowerUp SYBR Green Master Mix (Applied Biosystems, Foster City, CA, USA), on a 7900 HT Fast Real-Time PCR system RT-PCR (Applied Biosystems, Foster City, CA, USA). Primer oligos were purchased from Integrated DNA Technologies (Coralville, IA, USA). Primer sequences are shown in Table S6.

Long-term siRNA-based viability assays

For long-term cell viability assays in SUM159 cells, cells were treated with doxycycline (0.125 µg/ml) for 72 hours to induce HORMAD1 expression. Cells were then plated in 96-well plates (600 cells/well) and forward transfected with 25nM AllStar non-targeting siRNA (QIAGEN, Hilden, Germany) or targeting siRNAs (Dharmacon, Lafayette, CO, USA), using 0.1µL RNAiMax (Invitrogen, Thermo Fisher Scientific, Waltham, MA, USA) in a total volume of 100µL. Twenty-four hours after transfection, cells were passaged to 6-well plates in triplicate and grown for 11 days in the presence of doxycycline (0.125 µg/ml). Media containing fresh doxycycline was replaced every 72 hours. Cell viability was measured using CellTiter-Blue (Promega, Madison, WI, USA). Data are presented as surviving fraction, relative to mock-transfected cells. For clonogenic survival assays, SUM159, MCF10A and RPE-1 cells were treated with doxycycline (0.125 µg/ml, 0.5 µg/ml and 0.25 µg/ml for SUM159, MCF10A and RPE1, respectively) for 72 hours, plated in 96-well plates (700 cells/well and 400 cells/well respectively), forward transfected and passaged as described above. Cells were fixed with 10% trichloroacetic acid (Thermo Fisher Scientific, Waltham, MA, USA) and stained with sulphorhodamine B (Sigma-Aldrich, St. Louis, MO, USA). Colonies were counted manually. All cell-based assays were performed in triplicate wells and data shown is representative of 2 or more replicates.

siRNA mediated knockdown experiments in HORMAD1 positive cell lines

BT549, HCC38, HCC1143, MDA-MB-436 cells were plated in 96-well plates (1000 cells/well) and forward transfected with 25nM AllStar non-targeting siRNA (QIAGEN, Hilden, Germany) or targeting siRNAs (Dharmacon, Lafayette, CO, USA), using 0.1μL RNAiMax (Invitrogen, Thermo Fisher Scientific, Waltham, MA, USA) in a total volume of 100μL. Cell viability was estimated 7 days post transfection using CellTiter-Glo (Promega, Madison, WI, USA). Data were analysed and presented as normalised percentage inhibition (NPI).

CRISPR-Cas9-based viability assays

pLentiCRISPR-mCherry was a gift from Beat Bornhauser (Addgene plasmid # 75161, (7)). Edit-R crRNA sequences targeting POLH were custom-designed using CRISPR v4.2 software (TEFOR) (8). Target sequences of each crRNA are listed in Table S7. Stable Cas9-expressing cell lines were generated by lentivirus-mediated transduction of the pLentiCRISPR-mCherry construct, followed by FACS sorting of mCherry-positive cells using a BD FACSAria II flow cytometer (BD Biosciences, San Jose, CA, USA). For SUM159 experiments, cells were plated in 24-well plates and forward transfected with 10nM of Edit-R synthetic crRNA (Dharmacon, Lafayette, CO, USA) and 10nM of tracrRNA (Dharmacon, Lafayette, CO, USA) in a 1:1 ratio using 0.5μL RNAiMAX (Invitrogen, Thermo Fisher Scientific, Waltham, MA, USA), in a total volume of 500μL. After 24 hours, cells were passaged to 6-well plates in triplicate and grown for 11 days in the presence of doxycycline (0.125 μg/ml). Cell viability was measured using CellTiter-Blue (Promega, Madison, WI, USA). Data are presented as surviving fraction, relative to mock-transfected cells. For experiments in HCC38 and BT549, cells were plated in 96-well plates (1000 cells/well) and forward transfected with 10nM of Edit-R synthetic crRNA and 10nM of tracrRNA (Dharmacon, Lafayette, CO, USA)

using 0.05μL RNAiMAX (Invitrogen, Thermo Fisher Scientific, Waltham, MA, USA), in a total volume of 50μL. Cell proliferation was assessed by Incucyte (Sartorius, Göttingen, Germany) time-lapse within-incubator microscopy (4x zoom, 1 image per day) over a 6-day period, using the Cas9-mCherry signal as a marker of total nuclei number. Data are presented as the relative number of nuclei, normalised to mock-transfected cells. Assays were performed in triplicate wells and data shown is representative of 2 or more replicates.

Clonogenic dose-response survival assays

The ATR inhibitors VE-821, VX-970/M6620 (berzosertib), AZ20 and AZD6738 (ceralasertib) were purchased from Selleck Chemicals (Houston, TX, USA). For clonogenic dose-response survival assays, cells were seeded in 6-well plates at a density of 200 to 300 cells per well and continuously exposed to drug 16 hours after seeding, for 14 days. Media containing fresh drug was replaced every 72 hours. Cells were fixed with 10% trichloroacetic acid and stained with 0.4% sulphorhodamine B (SRB) (Sigma-Aldrich, St. Louis, MO, USA) in 1% acetic acid. SRB was solubilised with 10 mmol/L Tris-base (Sigma-Aldrich, St. Louis, MO, USA) and plates were read at 490 nm using a Wallac Victor 1420 multilabel counter (Perkin-Elmer, Waltham, MA, USA). Assays were performed in triplicate wells and data shown is representative of 2 or more replicates.

Western blot analysis

Cell lysates were prepared using 2% sodium dodecyl sulphate (SDS) (Invitrogen, Thermo Fisher Scientific, Waltham, MA, USA) and subsequently sonicated (2 cycles of 5 seconds, 80% amplitude). Cell lysates equivalent to 15-20μg of protein were diluted in 4x Laemmli sample buffer (277.8 mM Tris-HCl, pH6.8, 44.4% (v/v) glycerol, 4.4% SDS and 0.02% bromophenol blue), boiled for 5 minutes at 95°C and resolved

by SDS-PAGE gel electrophoresis. Proteins were transferred onto a nitrocellulose membrane using western blotting. Membranes were then blocked in 5% milk/TBS-T (20mM Tris, 150 mM NaCl, 0.1% Tween 20), and probed with primary antibody overnight at 4°C (diluted in 5% milk/TBS-T). The following primary antibodies were used in this study: HORMAD1 (1:500, HPA037850, Sigma-Aldrich, St. Louis, MO, USA), POLH (1:500, 13848, Cell Signaling, Danvers, MA, USA), Actin (1:30000, A5316, Sigma-Aldrich, St. Louis, MO, USA), Tubulin (1:5000, T5168, Sigma-Aldrich, St. Louis, MO, USA), Vinculin (1:5000, V9131, Sigma-Aldrich, St. Louis, MO, USA). Following primary antibody incubation, membranes were washed 4x with TBS-T for 5 minutes at room temperature, and then incubated with a secondary antibody (raised against the species of the primary antibody) conjugated to HRP. Membranes were washed a further 4 times for 5 minutes at room temperature before imaging using electrochemiluminescence (ECL) (GE Healthcare, Chicago, IL, USA).

Immunofluorescence analysis of γH2AX

Clonally-derived HORMAD1-inducible SUM159 cells were treated with doxycycline (0.125 μg/ml) for 48 hours, to induce HORMAD1 expression. Cells were then grown on Borosilicate Glass 22mm square coverslips (Fisher Scientific, Leicestershire, UK) for 24 hours in the presence of doxycycline (0.125 μg/ml) (72 hours total). Cells were fixed in 4% (v/v) PFA (Thermo Fisher Scientific, Waltham, MA, USA) and permeabilised with 0.2% (v/v) Triton X-100 (Sigma-Aldrich, St. Louis, MO, USA) in PBS for 10 minutes at room temperature. Coverslips were incubated with DNase I (Sigma-Aldrich, St. Louis, MO, USA) diluted to 1000 units/ml in PBS, for 2 hours in a humidified chamber at 37°C. Cells were immunostained with primary antibody against γH2AX (05-636, Merck Millipore, Burlington, MA, USA) diluted 1:1000 in IFF buffer (1% bovine serum albumin, 2% foetal calf serum in PBS). Cells were washed 3x with

PBS, incubated with fluorescently labelled anti-mouse Alexa Fluor® 555 (Invitrogen, Thermo Fisher Scientific, Waltham, MA, USA) secondary antibody for 1 hour at room temperature, washed another 3x with PBS, and then counterstained with 1µg/mL DAPI (Invitrogen, Thermo Fisher Scientific, Waltham, MA, USA). Coverslips were mounted onto microscope slides using Vectashield antifade mounting medium (Vector Laboratories Inc., Burlingame, CA, USA) and imaged at 100x on a Nikon A1 confocal microscope (Nikon, Tokyo, Japan). Data was quantified as percentage of DAPI-stained nuclei with >5 γH2AX foci. Approximately 150 cells were scored per condition per replicate and 4 replicates were performed.

Aberrant nuclear structure analysis

Clonally-derived HORMAD1(or GFP)-inducible SUM159 cells were passaged in continuous presence of doxycycline (0.125 µg/ml) for 8 weeks. Cells were then seeded on Borosilicate Glass 22mm coverslips (Fisher Scientific, Leicestershire, UK) and grown for 24 hours. Cells were fixed in 4% (v/v) PFA (Thermo Fisher Scientific, Waltham, MA, USA), permeabilised in Triton X-100 (0.2%) and stained with 1µg/mL DAPI (Thermo Fisher Scientific, Waltham, MA, USA). Slides were analysed by eye at 100x on a Zeiss fluorescent microscope (Carl Zeiss, Oberkochen, Germany). Approximately 150 cells were scored for aberrant nuclear structures (micronuclei, nuclear buds and anaphase bridges) per condition, per replicate and 4 replicates were performed.

STRING Protein Network analysis

STRING Protein Network Analysis (9) was carried out by importing the list of 63 candidate genes into the multiple proteins search tab and selecting *Homo sapiens* as the organism. MCL clustering with inflation parameter of 3 was used as the clustering method. For network settings, the following settings were used: 1) meaning of network

edges: evidence; 2) active interaction sources: textmining, experiments, databases, co-expression, neighborhood, gene fusion and co-occurrence; 3) minimum required interaction score: medium confidence (0.400); 4) network display mode: interactive svg.

Statistical analysis

All data are presented as the mean with standard deviation, from 3 replicate experiments. All pair-wise statistical analysis was performed using parametric t-tests, unless otherwise stated.

Supplementary Figures

Supplementary Figure Legends

Fig. S1: Doxycycline-inducible HORMAD1 expression drives genomic instability. A Western blot analysis of doxycycline-induced HORMAD1 expression in two clonally-derived HORMAD1-inducible SUM159 cell lines. Asterisk refers to a non-specific band. **B** Western blot analysis of doxycycline-induced HORMAD1 expression in isogenic cell line models compared expression in the HORMAD1 positive cell line MDAMB436. **C** Scatter dot plot displaying increased percentage of cells with >5 γH2AX foci per DAPI-stained nucleus, measured after acute expression of HORMAD1 (72 hours). Approximately 150 cells were analysed per condition. Each dot represents a single biological replicate (*n*= 4). *p* values represent Fisher's exact test. **D** Representative immunofluorescence images of γH2AX foci. **E** Scatter dot plot displaying increased percentage of aberrant nuclear structures (micronuclei, nuclear buds, nucleoplasmic bridges) per DAPI-stained nucleus measured 8 weeks after continuous expression of HORMAD1. Approximately 150 cells were analysed per condition. Each dot represents a single biological replicate (*n*= 4). *p* values represent Fisher's exact test. **F** Representative immunofluorescence images of aberrant nuclear structures. **G** Flow cytometry analysis of doxycycline-induced GFP expression in two clonally-derived GFP-inducible SUM159 cell lines. **H** Scatter dot plot displaying no significant changes in the percentage of aberrant nuclear structures (micronuclei, nuclear buds, nucleoplasmic bridges) per DAPI-stained nucleus measured 8 weeks after continuous expression of GFP. Approximately 150 cells were analysed per condition. Each dot represents a single biological replicate (*n*= 4). *p* values represent Fisher's exact test.

Fig. S2: RNAi screen quality control data. A-B Representative Z' factor analysis showing the distribution of positive (siPLK1) and negative control (siCON1, siCON2, ALLSTAR) Z-score values. A Z' factor value >0.3 indicates a good separation of positive and negative control Z-score values. **C** Boxplot illustrating the distribution of Z-scores of positive and negative control siRNAs for each technical replicate in the clonally-derived HORMAD1-inducible SUM159 doxycycline experimental arm. Boxes represent individual Z-scores and error bars represent the SD. **D** Representative Spearman's correlation analysis of Z-scores from replicate 1 and 2 in the clonally-derived HORMAD1-inducible SUM159 doxycycline experimental arm (r^2= 0.96).

Fig. S3: Fourteen DDR-associated gene candidates identified in the primary screen. A-N Bar plots displaying Z-scores (*grey*) and Drug Effect (DE) Z-scores (*red*= clonally-derived HORMAD1-inducible SUM159, *black*= parental SUM159) for the 14 DDR-associated gene candidates identified in the primary screen. siRNAs that significantly impaired cell viability following HORMAD1 expression were identified on the basis of criteria discussed in text.

Fig. S4: Validation of siRNA gene silencing effects by RT-qPCR analysis. A-E Bar plot displaying the percentage of mRNA expression of the indicated gene, following transfection of clonally-derived HORMAD1-inducible SUM159 with an siRNA SMARTpool or 4 individual siRNAs targeting: **A** *ATR;* **B** *BRIP1*; **C** *POLH;* **D** *TDP1*; **E** *XRCC1.* Non-targeting siRNA (siALLSTAR) was used as a control. Gene silencing effects were evaluated 48 hours post-transfection by RT-qPCR, using primers specific towards the respective gene and *ACTB* as a normalisation control. Error bars indicate SD of the mean (*n*= 3) and *p* values represent the Student's t test.

Fig. S5: Deconvolution RNAi validation of 14 gene candidates. A-J Bar plots displaying normalised percentage inhibition (NPI) of clonally-derived control GFP-

inducible SUM159 cells (+DOX/+GFP *vs.* -DOX/-GFP) or parental SUM159 cells, transfected with siRNA SMARTpool or four individual siRNAs targeting **A-B** *ATR*; **C-D** *BRIP1*; **E-F** *POLH*; **G-H** *TDP1*; **I-J** *XRCC1* and exposed to GFP expression or doxycycline treatment for 4 days. Non-targeting (siALLSTAR) and targeting (siPLK1) siRNAs were used as normalisation controls. Fourteen DDR-related gene candidates were further evaluated in secondary deconvolution experiments. *ATR*, *BRIP1*, *POLH*, *TDP1* and *XRCC1* met the validation criteria discussed in text. Error bars indicate SD from mean effects, *p* values represent multiple Student t tests (*p*= ***< 0.0001, *p*= **< 0.001, *p*= *<0.05).

Fig. S6: Generation of clonally-derived HORMAD1-inducible MCF10A and RPE1 cell lines. A-B Western blot analysis of doxycycline-induced HORMAD1 expression in **A** clonally-derived HORMAD1-inducible MCF10A and **B** clonally-derived HORMAD1-inducible RPE1. **C-D** Cell growth curves displaying reduced cell number of **C** MCF10A cells and **D** RPE1 cells, following acute expression of HORMAD1 (48 hours). Cell number was normalised relative to T0 counts. Error bars indicate SD from mean effects (*n*= 3), *p* values represent multiple Student t tests. **E** and **F** Comparison of induced HORMAD1 expression to that of endogenous levels in MDAMB436.

Fig. S7: HORMAD1-associated genetic dependencies in HORMAD1 positive cell lines. Candidate HORMAD1 genetic dependencies were knocked down using siRNA in four HORMAD1 positive cell lines. **A-C** and **G-H** Show effect of knockdown on cell viability 7 days post transfection. Data is presented as normalised percent inhibition (NPI). Data from triplicate wells are shown and error bars indicate SD from mean, *p* values represent Student t tests. **D-F and I-J** Show knockdown achieved by siRNA (NT black and siRNA red) in each cell line. Knockdown was measured by RT-qPCR

and normalised to *ACTB*. Data from triplicate qPCR reactions are shown and error bars indicate SD from mean.

Fig. S8: HORMAD1 expression does not sensitise cells to ATR inhibitors. A-D Clonogenic dose-response survival curves displaying no significant changes in cell survival of HORMAD1-inducible SUM159 cells (+DOX/+HORMAD1 *vs.* -DOX/-HORMAD1) exposed to increasing concentrations of **A** VE-821, **B** VX970, **C** AZ20 and **D** AZD6738 for 14 days. Error bars indicate SD from mean effects (*n*= 3), *p* values represent two-way repeated measures ANOVA. **E-F** Clonogenic dose-response survival curves displaying no significant changes in cell survival of **E** HORMAD1-inducible MCF10A cells (+DOX/+HORMAD1 *vs.* -DOX/-HORMAD1) and **F** HORMAD1-inducible RPE1 cells (+DOX/+HORMAD1 *vs.* -DOX/-HORMAD1) exposed to increasing concentrations of AZD6738 for 14 days. Error bars indicate SD from mean effects (*n*= 3), *p* values represent two-way repeated measures ANOVA.

Fig. S9: Validation of HORMAD1-driven POLH dependency in 21.5-weeks HORMAD1-expressing SUM159 cells.

A Bar plot displaying reduced surviving fractions of clonally-derived HORMAD1-inducible SUM159 cells (+DOX/+HORMAD1 *vs.* -DOX/-HORMAD1) previously exposed to HORMAD1 expression for 20 weeks, transfected with siRNAs targeting *POLH* and exposed to HORMAD1 expression for 11 additional days. Non-targeting (siALLSTAR) and targeting (siPLK1) siRNAs were used as transfection controls and surviving fractions calculated from mock-transfected cells. Error bars indicate SD from mean effects (*n*= 3), *p* values represent multiple Student t tests (*p*= ***< 0.0001, *p*= **< 0.001, *p*= *<0.05). **B** Bar plot displaying the percentage of *POLH* mRNA expression following siRNA-mediated depletion of *POLH* described in **A**, measured by

RT-qPCR and normalised to *ACTB*.

Fig. S10: Candidate hits lacking HORMAD1-driven selectivity in SUM159, MCF10A and RPE1 cells. A Bar plot displaying no significant changes in colony counts of SUM159 cells (+DOX/+HORMAD1 *vs.* -DOX/-HORMAD1) transfected with siRNA SMARTpools targeting *POLK, POLI* and *REV1*. Non-targeting (siALLSTAR) siRNA was used as normalisation control. Error bars indicate SD from mean effects (*n*= 3), *p* values represent multiple Student t tests. **B** Representative colony images from experiment **A**. **C** Bar plot displaying the percentage of *POLK*, *POLI* and *REV1* mRNA expression following siRNA-mediated gene knockdown for experiments described in **A**, measured by RT-qPCR and normalised to *ACTB*. **D** Bar plot displaying no significant changes in colony counts of MCF10A cells (+DOX/+HORMAD1 *vs.* -DOX/-HORMAD1) transfected with siRNA SMARTpools targeting *POLI* and *REV3L*. Non-targeting (siALLSTAR) siRNA was used as normalisation control. Error bars indicate SD from mean effects (*n*= 3), *p* values represent multiple Student t tests. **E** Representative colony images from experiment **D. F** Bar plot displaying the percentage of *POLI* and *REV3L* mRNA expression following siRNA-mediated gene knockdown for experiments described in **D**, measured by RT-qPCR and normalised to *ACTB*. **G** Bar plot displaying no significant changes in colony counts of RPE1 cells (+DOX/+HORMAD1 *vs.* -DOX/-HORMAD1) transfected with siRNA SMARTpools targeting *POLI* and *REV1*. Non-targeting (siALLSTAR) siRNA was used as normalisation control. Error bars indicate SD from mean effects (*n*= 3), *p* values represent multiple Student t tests. **H** Representative colony images from experiment **G. I** Bar plot displaying the percentage of *POLI* and *REV1* mRNA expression following siRNA-mediated gene knockdown for experiments described in **G**, measured by RT-qPCR and normalised to *ACTB*.

Fig. S11: HORMAD1-associated TLS genetic dependencies in HORMAD1 positive cell lines. HORMAD1-associated genetic dependencies in HORMAD1 positive cell lines. Candidate HORMAD1 genetic dependencies were knocked down using siRNA in four HORMAD1 positive cell lines. **A-B and E-F** Show effect of knockdown on cell viability 7 days post transfection. Data is presented as normalised percent inhibition (NPI). Data from triplicate wells are shown and error bars indicate SD from mean, *p* values represent multiple Student t tests. **C-D and G-H** Show knockdown achieved by siRNA in each cell line (NT black and siRNA red). Knockdown was measured by RT-qPCR and normalised to *ACTB*. Data from triplicate qPCR reactions are shown and error bars indicate SD from mean.

Fig. S12 – Fig. S17: Uncropped scans of western blots.

Fig. S1

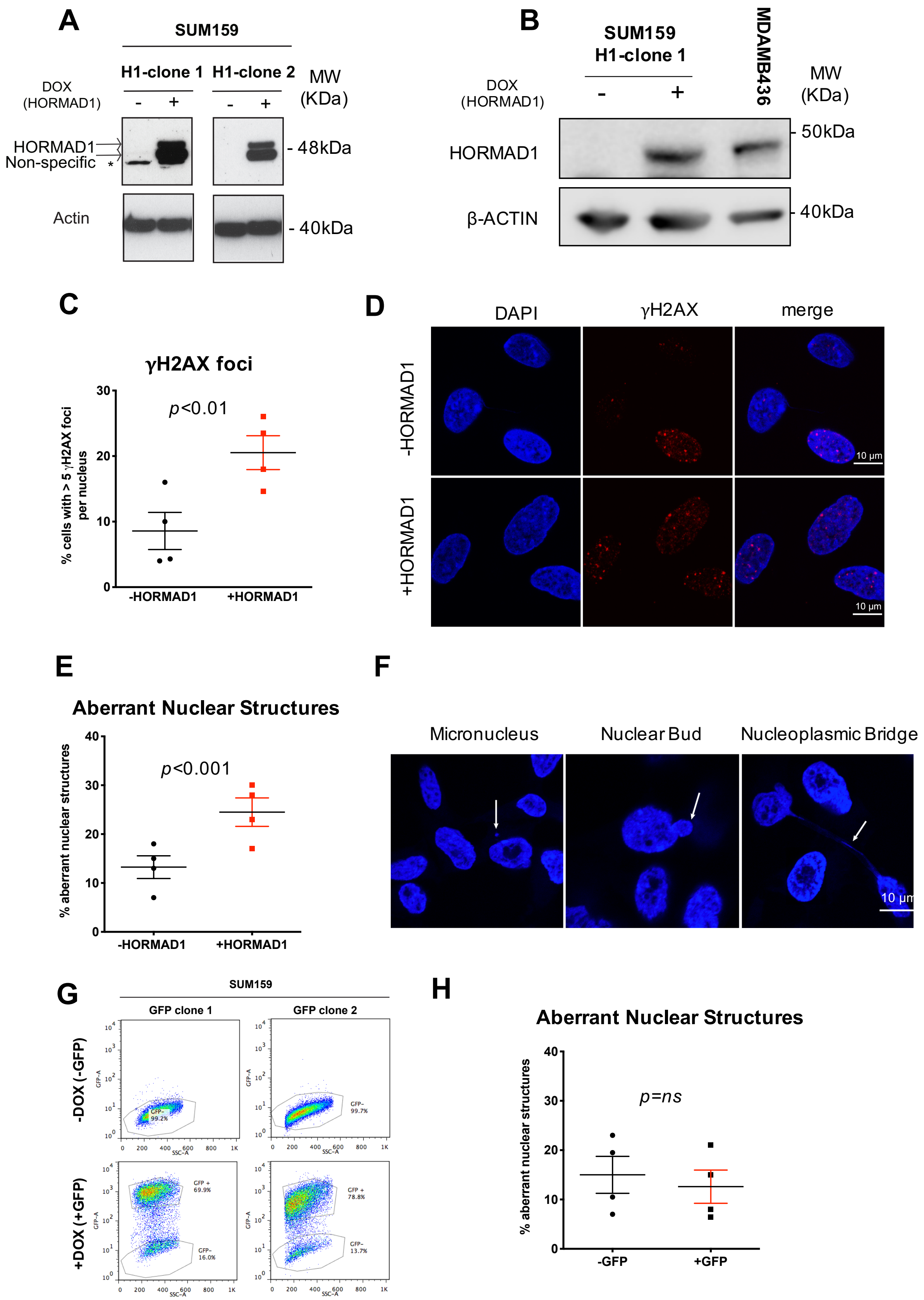
A
SUM159
H1-clone 1
H1-clone 2
MW (KDa)
DOX (HORMAD1)
HORMAD1
Non-specific
48kDa
Actin
40kDa
B
SUM159 H1-clone 1
MDAMB436
MW (KDa)
DOX (HORMAD1)
HORMAD1
50kDa
β-ACTIN
40kDa
C
γH2AX foci
p<0.01
% cells with > 5 γH2AX foci per nucleus
-HORMAD1
+HORMAD1
D
DAPI
γH2AX
merge
-HORMAD1
+HORMAD1
10 µm
E
Aberrant Nuclear Structures
p<0.001
% aberrant nuclear structures
-HORMAD1
+HORMAD1
F
Micronucleus
Nuclear Bud
Nucleoplasmic Bridge
G
SUM159
GFP clone 1
GFP clone 2
-DOX (-GFP)
+DOX (+GFP)
H
Aberrant Nuclear Structures
p=ns
% aberrant nuclear structures
-GFP
+GFP

Fig. S2

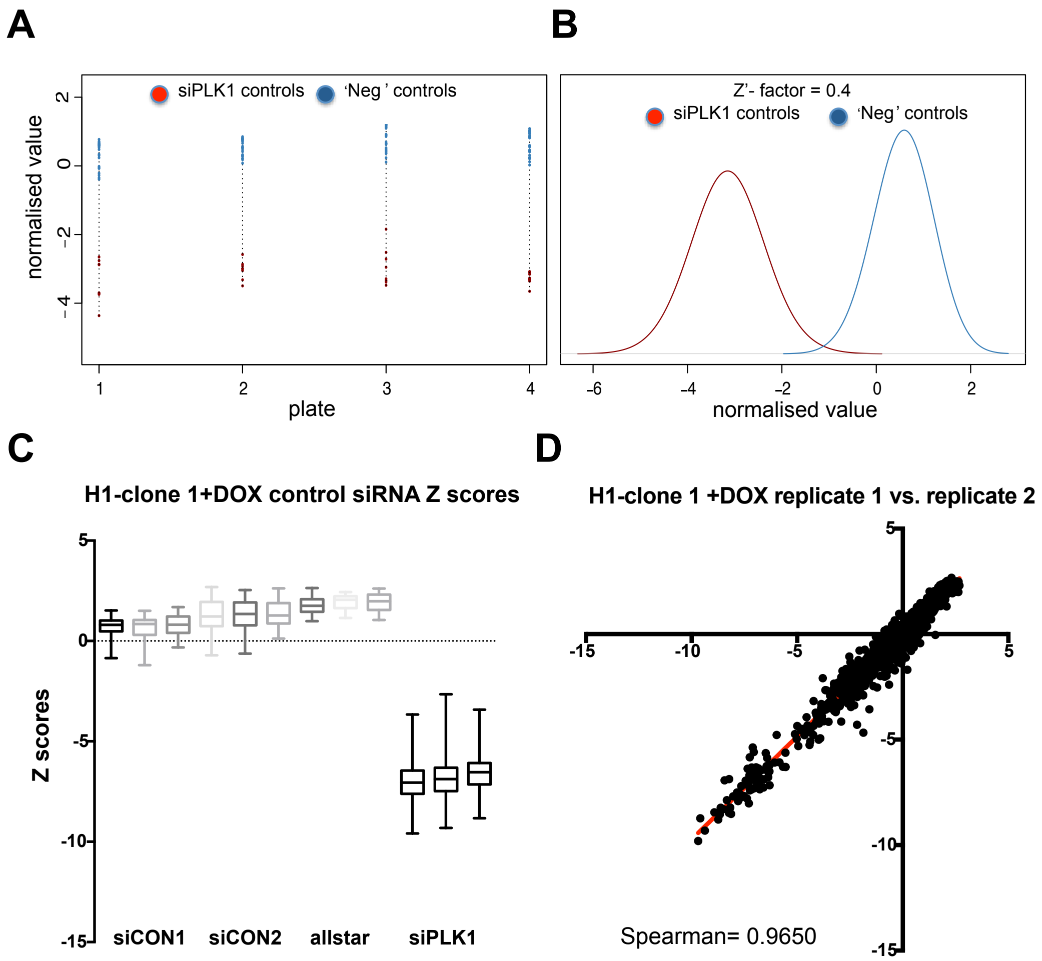
A
siPLK1 controls
'Neg' controls
normalised value
plate
B
Z'- factor = 0.4
siPLK1 controls
'Neg' controls
normalised value
C
H1-clone 1+DOX control siRNA Z scores
Z scores
siCON1
siCON2
allstar
siPLK1
D
H1-clone 1 +DOX replicate 1 vs. replicate 2
Spearman= 0.9650

Fig. S3

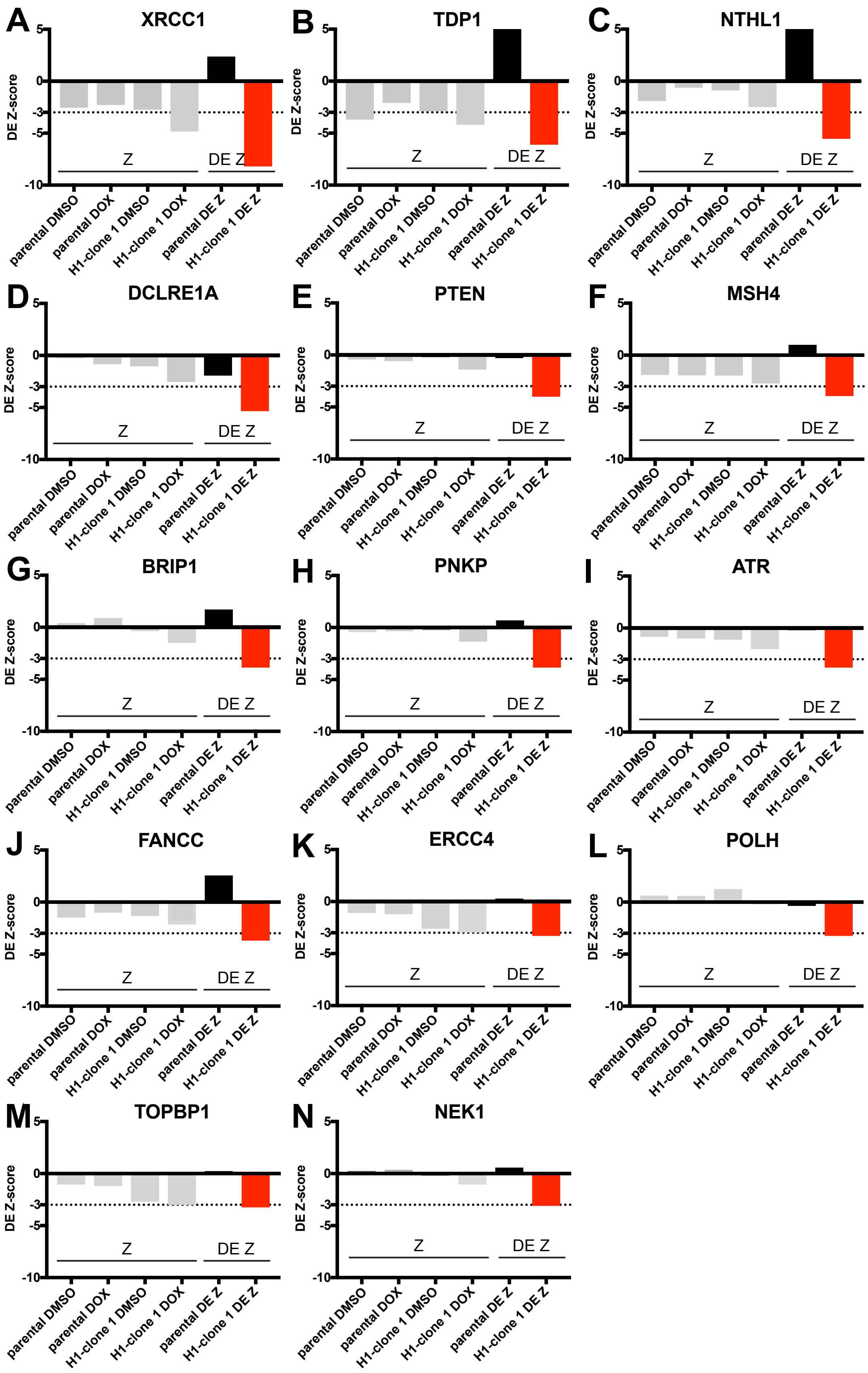
A
XRCC1
B
TDP1
C
NTHL1
D
DCLRE1A
E
PTEN
F
MSH4
G
BRIP1
H
PNKP
I
ATR
J
FANCC
K
ERCC4
L
POLH
M
TOPBP1
N
NEK1
DE Z-score
5
0
-3
-5
-10
Z
DE Z
parental DMSO
parental DOX
H1-clone 1 DMSO
H1-clone 1 DOX
parental DE Z
H1-clone 1 DE Z

Fig. S4

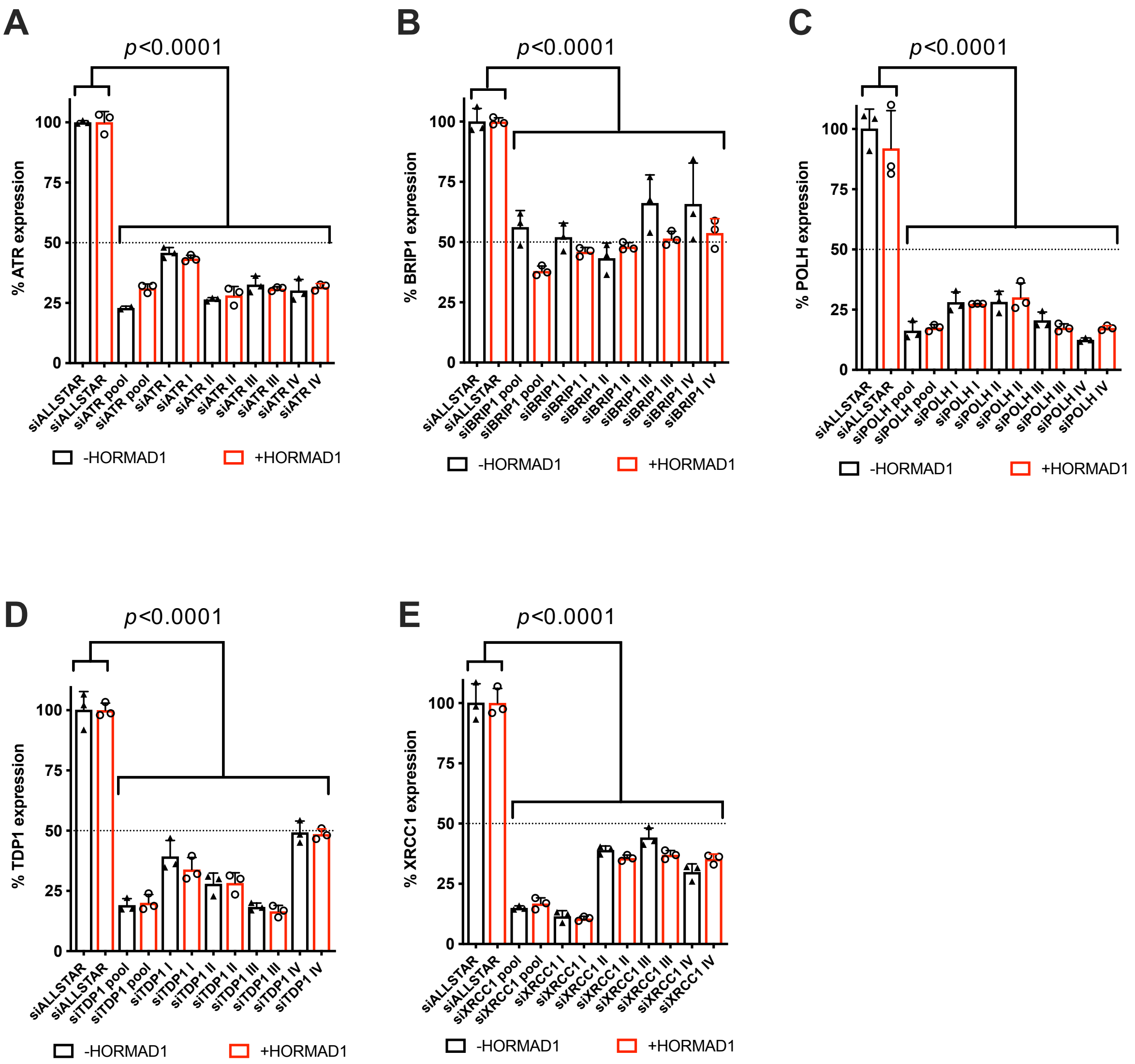
A
p<0.0001
% ATR expression
0
25
50
75
100
siALLSTAR
siALLSTAR
siATR pool
siATR pool
siATR I
siATR I
siATR II
siATR II
siATR III
siATR III
siATR IV
siATR IV
-HORMAD1
+HORMAD1
B
p<0.0001
% BRIP1 expression
siALLSTAR
siALLSTAR
siBRIP1 pool
siBRIP1 pool
siBRIP1 I
siBRIP1 I
siBRIP1 II
siBRIP1 II
siBRIP1 III
siBRIP1 III
siBRIP1 IV
siBRIP1 IV
-HORMAD1
+HORMAD1
C
p<0.0001
% POLH expression
siALLSTAR
siALLSTAR
siPOLH pool
siPOLH pool
siPOLH I
siPOLH I
siPOLH II
siPOLH II
siPOLH III
siPOLH III
siPOLH IV
siPOLH IV
-HORMAD1
+HORMAD1
D
p<0.0001
% TDP1 expression
siALLSTAR
siALLSTAR
siTDP1 pool
siTDP1 pool
siTDP1 I
siTDP1 I
siTDP1 II
siTDP1 II
siTDP1 III
siTDP1 III
siTDP1 IV
siTDP1 IV
-HORMAD1
+HORMAD1
E
p<0.0001
% XRCC1 expression
siALLSTAR
siALLSTAR
siXRCC1 pool
siXRCC1 pool
siXRCC1 I
siXRCC1 I
siXRCC1 II
siXRCC1 II
siXRCC1 III
siXRCC1 III
siXRCC1 IV
siXRCC1 IV
-HORMAD1
+HORMAD1

Fig. S5

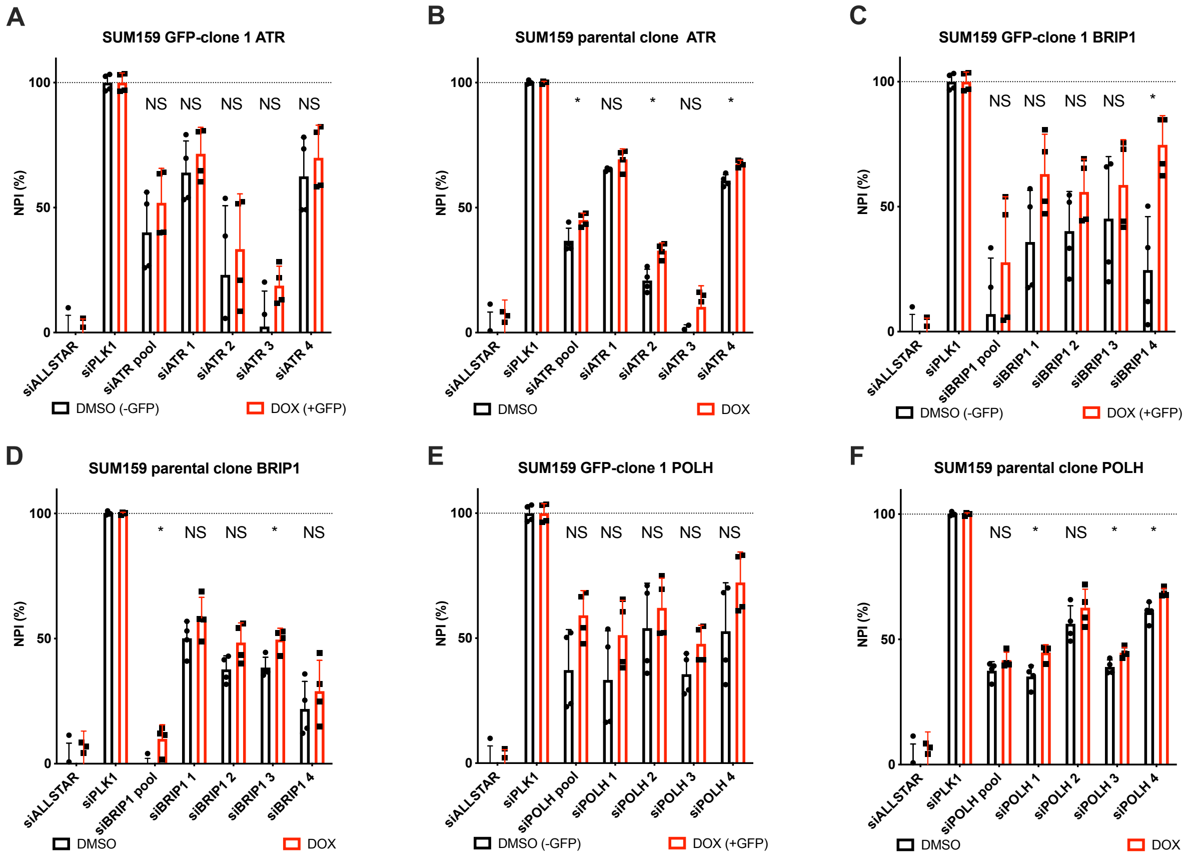

A
SUM159 GFP-clone 1 ATR
NPI (%)
NS NS NS NS NS
siALLSTAR
siPLK1
siATR pool
siATR 1
siATR 2
siATR 3
siATR 4
DMSO (-GFP)
DOX (+GFP)
B
SUM159 parental clone ATR
NPI (%)
* NS * NS *
siALLSTAR
siPLK1
siATR pool
siATR 1
siATR 2
siATR 3
siATR 4
DMSO
DOX
C
SUM159 GFP-clone 1 BRIP1
NPI (%)
NS NS NS NS *
siALLSTAR
siPLK1
siBRIP1 pool
siBRIP1 1
siBRIP1 2
siBRIP1 3
siBRIP1 4
DMSO (-GFP)
DOX (+GFP)
D
SUM159 parental clone BRIP1
NPI (%)
* NS NS * NS
siALLSTAR
siPLK1
siBRIP1 pool
siBRIP1 1
siBRIP1 2
siBRIP1 3
siBRIP1 4
DMSO
DOX
E
SUM159 GFP-clone 1 POLH
NPI (%)
NS NS NS NS NS
siALLSTAR
siPLK1
siPOLH pool
siPOLH 1
siPOLH 2
siPOLH 3
siPOLH 4
DMSO (-GFP)
DOX (+GFP)
F
SUM159 parental clone POLH
NPI (%)
NS * NS * *
siALLSTAR
siPLK1
siPOLH pool
siPOLH 1
siPOLH 2
siPOLH 3
siPOLH 4
DMSO
DOX

Fig. S5
continued

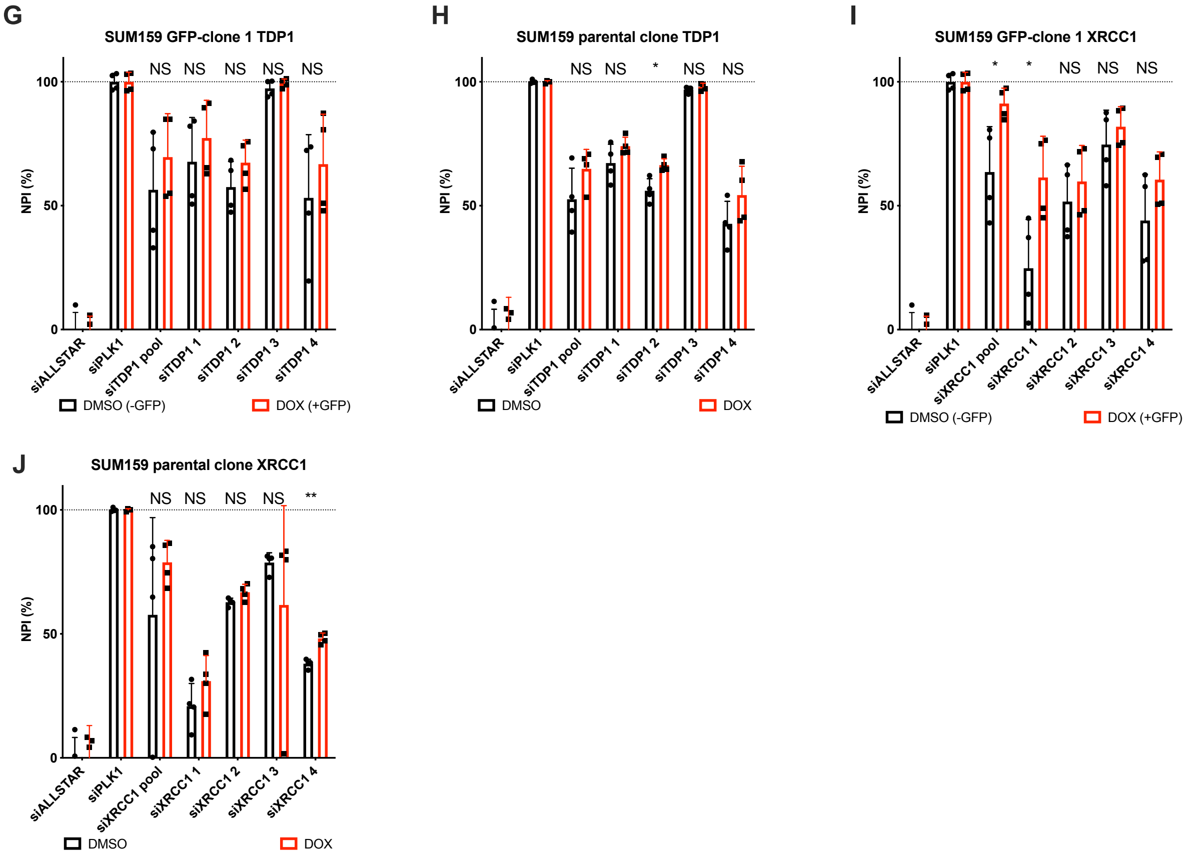

Fig. S6

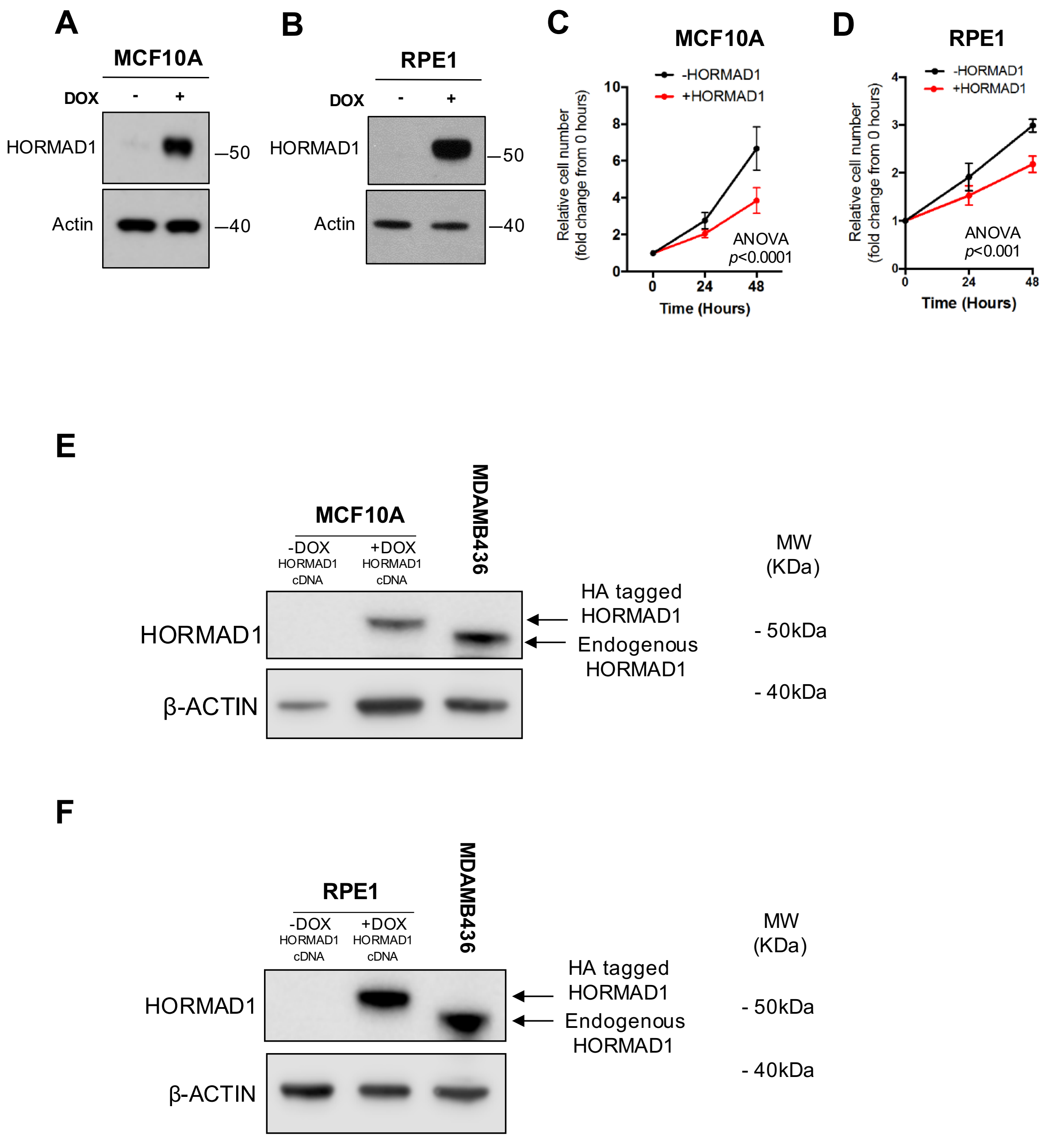

A
MCF10A
DOX
-
+
HORMAD1
—50
Actin
—40
B
RPE1
DOX
-
+
HORMAD1
—50
Actin
—40
C
MCF10A
-HORMAD1
+HORMAD1
Relative cell number
(fold change from 0 hours)
Time (Hours)
ANOVA
p<0.0001
D
RPE1
-HORMAD1
+HORMAD1
Relative cell number
(fold change from 0 hours)
Time (Hours)
ANOVA
p<0.001
E
MCF10A
-DOX HORMAD1 cDNA
+DOX HORMAD1 cDNA
MDAMB436
MW (KDa)
HORMAD1
β-ACTIN
HA tagged HORMAD1
Endogenous HORMAD1
- 50kDa
- 40kDa
F
RPE1
-DOX HORMAD1 cDNA
+DOX HORMAD1 cDNA
MDAMB436
MW (KDa)
HORMAD1
β-ACTIN
HA tagged HORMAD1
Endogenous HORMAD1
- 50kDa
- 40kDa

Fig. S7

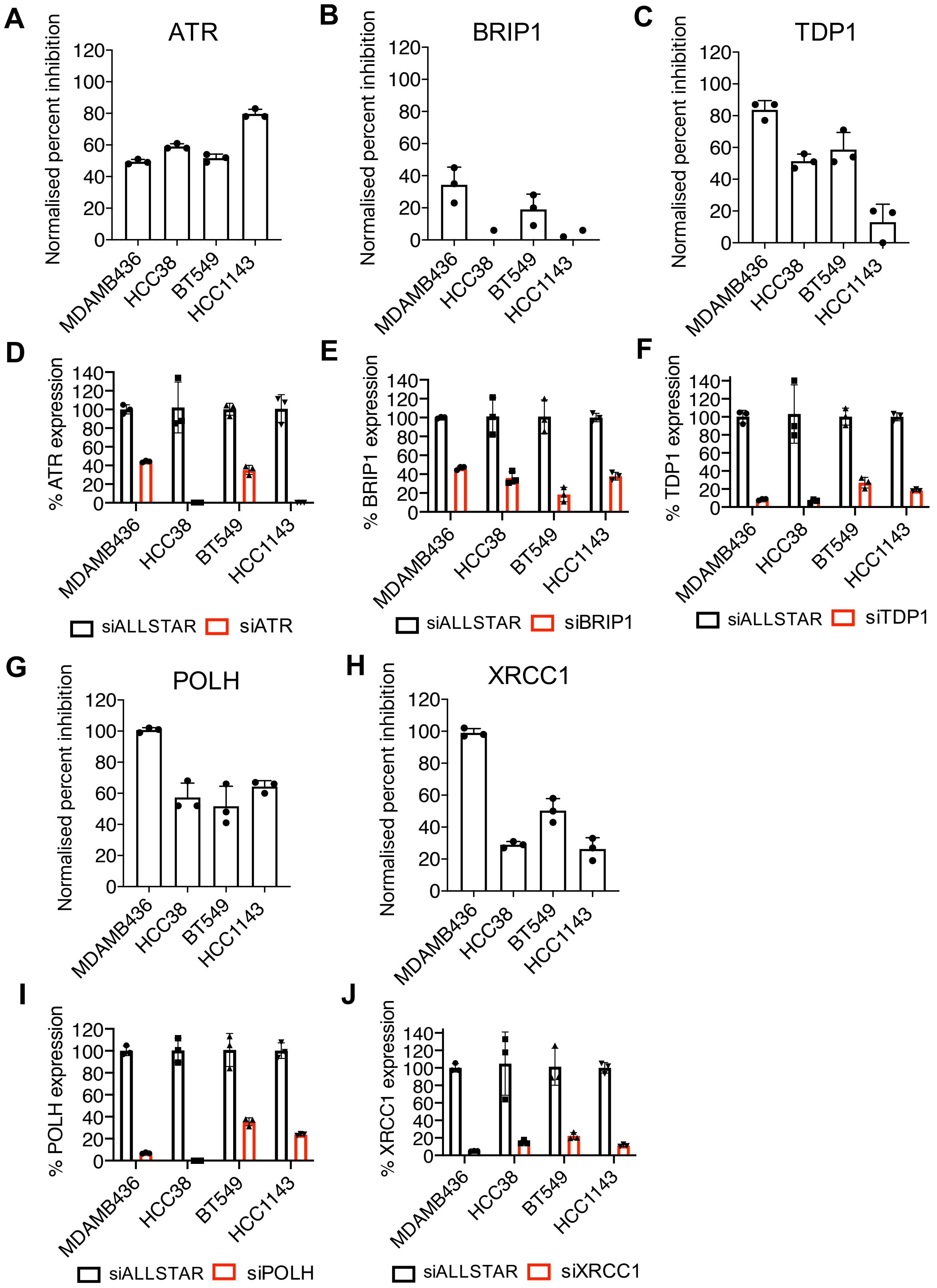
A
ATR
Normalised percent inhibition
0
20
40
60
80
100
120
MDAMB436
HCC38
BT549
HCC1143
B
BRIP1
Normalised percent inhibition
C
TDP1
Normalised percent inhibition
D
% ATR expression
140
siALLSTAR
siATR
E
% BRIP1 expression
siBRIP1
F
% TDP1 expression
siTDP1
G
POLH
Normalised percent inhibition
H
XRCC1
Normalised percent inhibition
I
% POLH expression
siPOLH
J
% XRCC1 expression
siXRCC1

Fig. S8

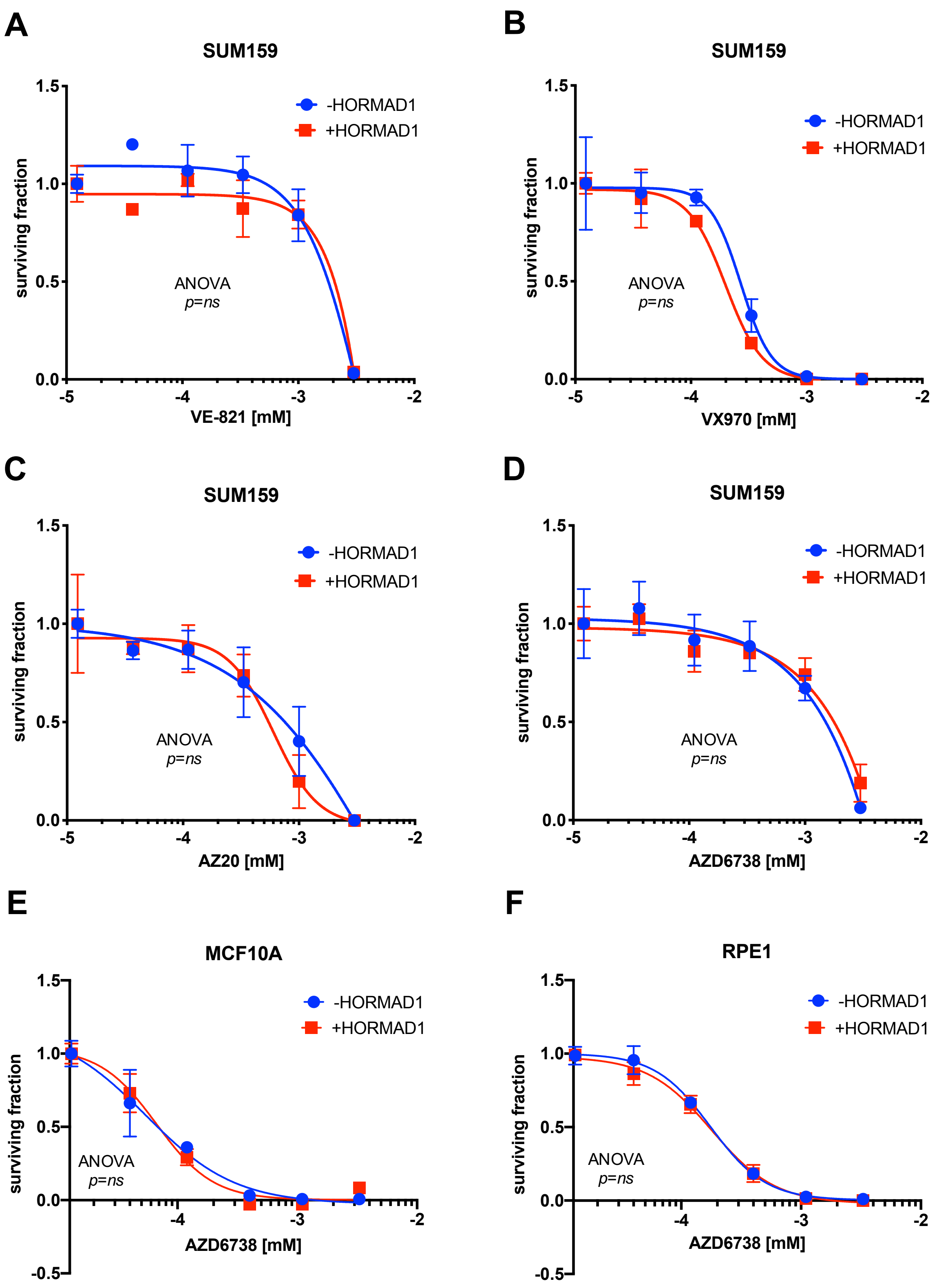
A
SUM159
-HORMAD1
+HORMAD1
surviving fraction
ANOVA
p=ns
VE-821 [mM]
B
SUM159
VX970 [mM]
C
SUM159
AZ20 [mM]
D
SUM159
AZD6738 [mM]
E
MCF10A
AZD6738 [mM]
F
RPE1
AZD6738 [mM]

Fig. S9

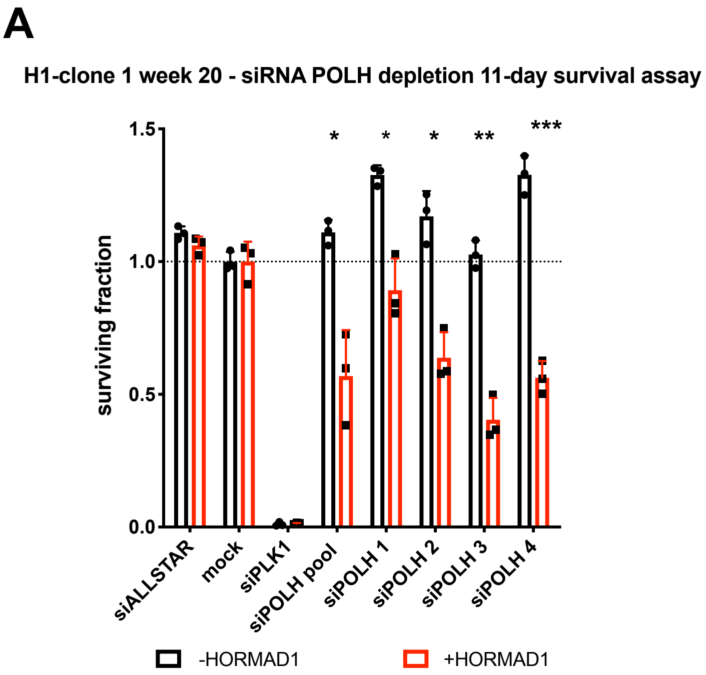
A
H1-clone 1 week 20 - siRNA POLH depletion 11-day survival assay
surviving fraction
1.5
1.0
0.5
0.0
*
*
*
**
***
siALLSTAR
mock
siPLK1
siPOLH pool
siPOLH 1
siPOLH 2
siPOLH 3
siPOLH 4
-HORMAD1
+HORMAD1

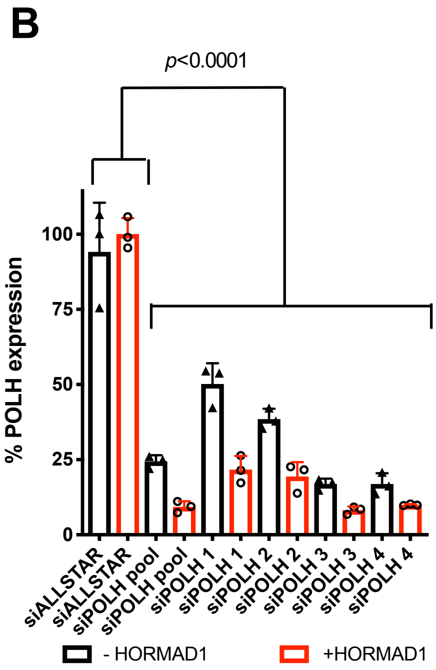
B
p<0.0001
% POLH expression
100
75
50
25
0
siALLSTAR
siALLSTAR
siPOLH pool
siPOLH pool
siPOLH 1
siPOLH 1
siPOLH 2
siPOLH 2
siPOLH 3
siPOLH 3
siPOLH 4
siPOLH 4
- HORMAD1
+HORMAD1

Fig. S10

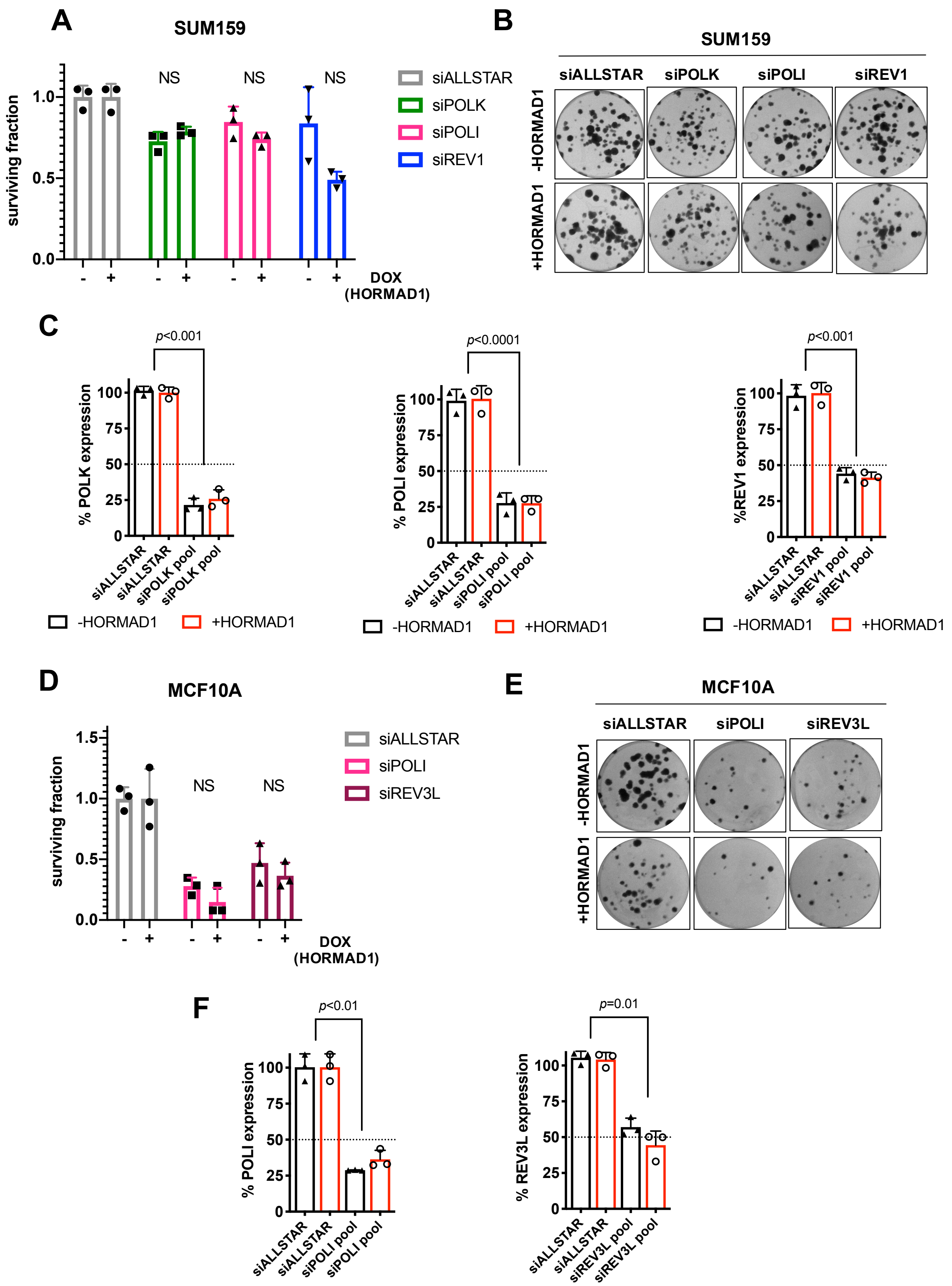
A
SUM159
surviving fraction
NS
siALLSTAR
siPOLK
siPOLI
siREV1
DOX
(HORMAD1)
B
SUM159
siALLSTAR
siPOLK
siPOLI
siREV1
-HORMAD1
+HORMAD1
C
p<0.001
% POLK expression
siALLSTAR
siPOLK pool
p<0.0001
% POLI expression
siPOLI pool
%REV1 expression
siREV1 pool
-HORMAD1
+HORMAD1
D
MCF10A
siREV3L
E
MCF10A
F
p<0.01
p=0.01
% REV3L expression
siREV3L pool

Fig. S10 *continued*

G

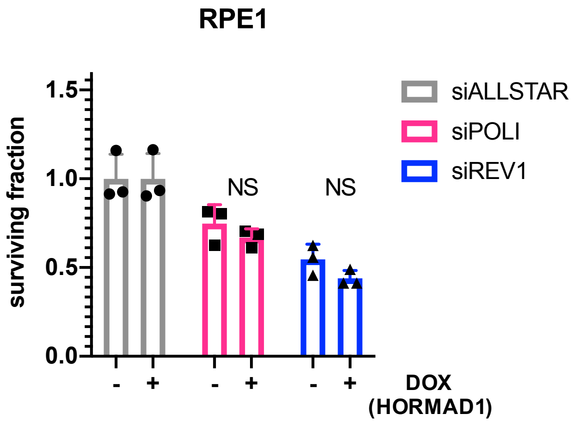

H

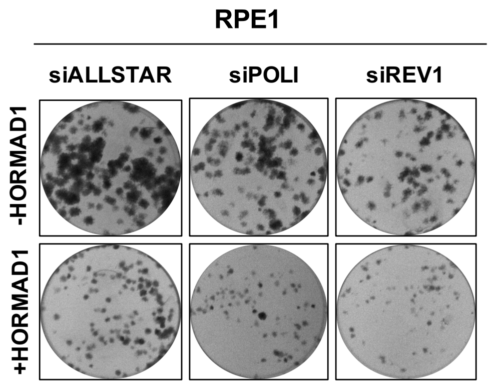

I

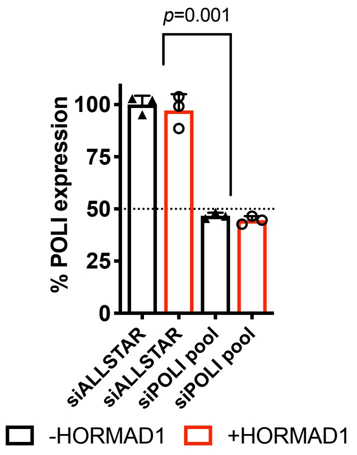

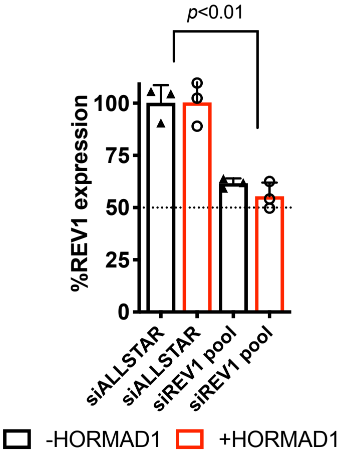

Fig. S11

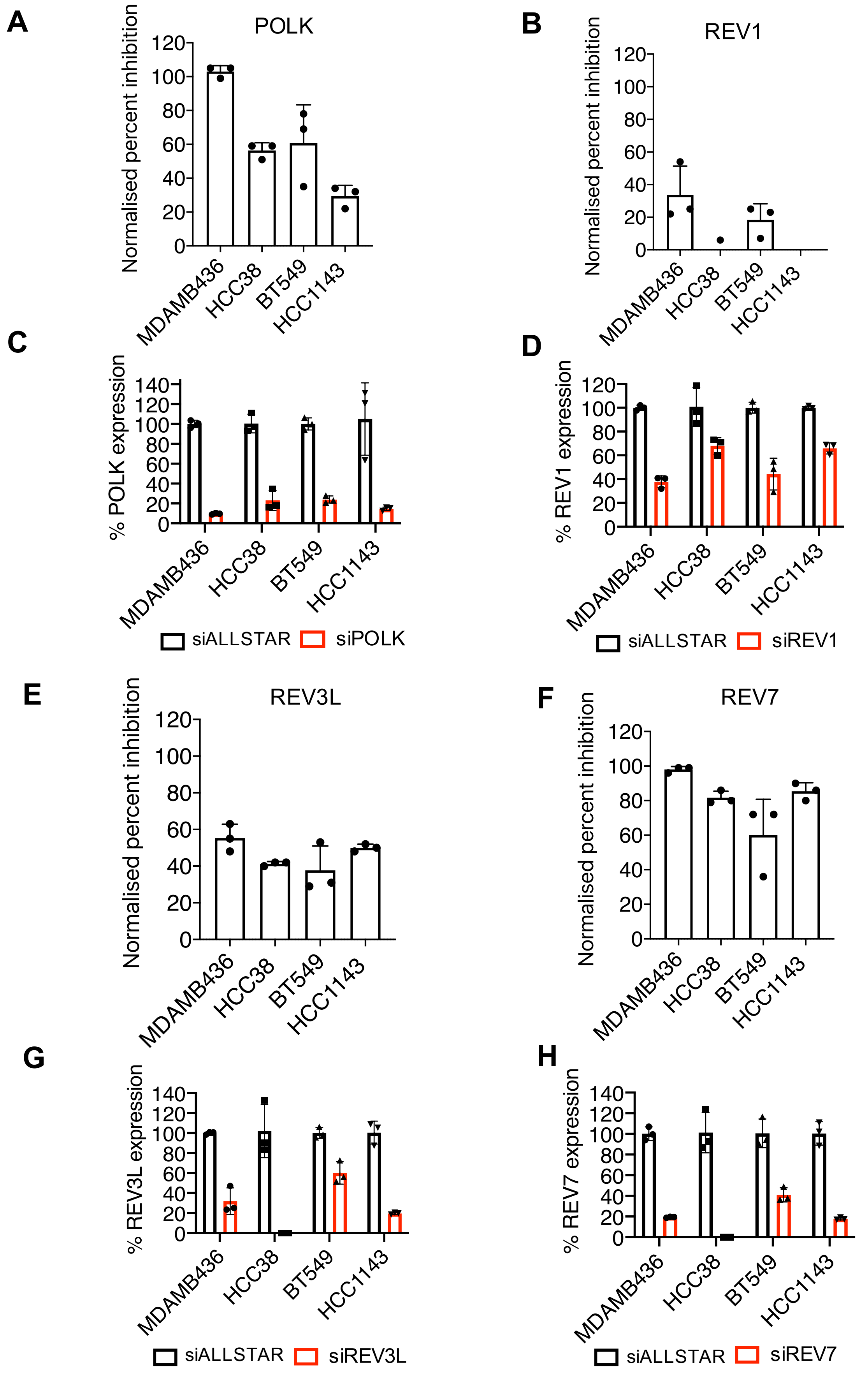
A
POLK
Normalised percent inhibition
120
100
80
60
40
20
0
MDAMB436
HCC38
BT549
HCC1143
B
REV1
Normalised percent inhibition
C
% POLK expression
140
siALLSTAR
siPOLK
D
% REV1 expression
siREV1
E
REV3L
F
REV7
G
% REV3L expression
siREV3L
H
% REV7 expression
siREV7

Fig. S12

A

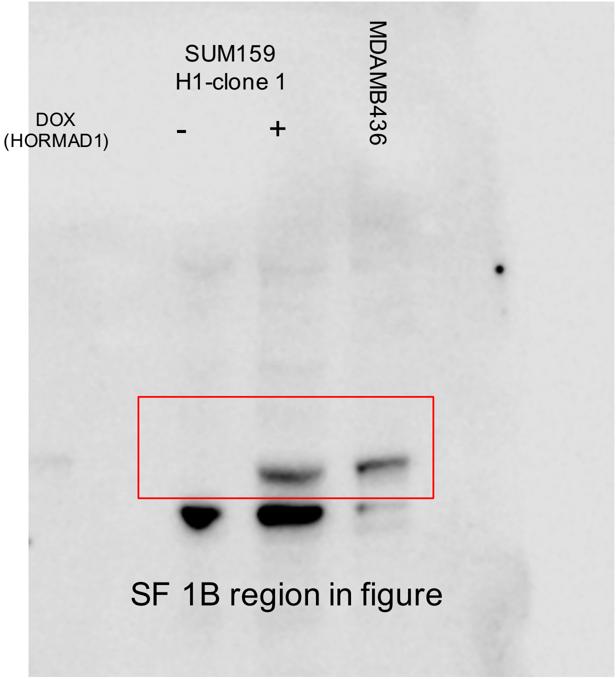

Anti-HORMAD1

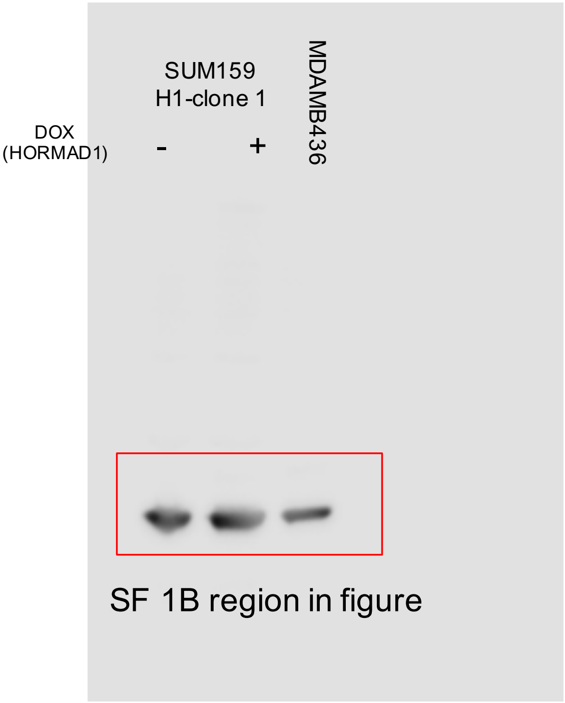

Anti-Actin

Fig. S13

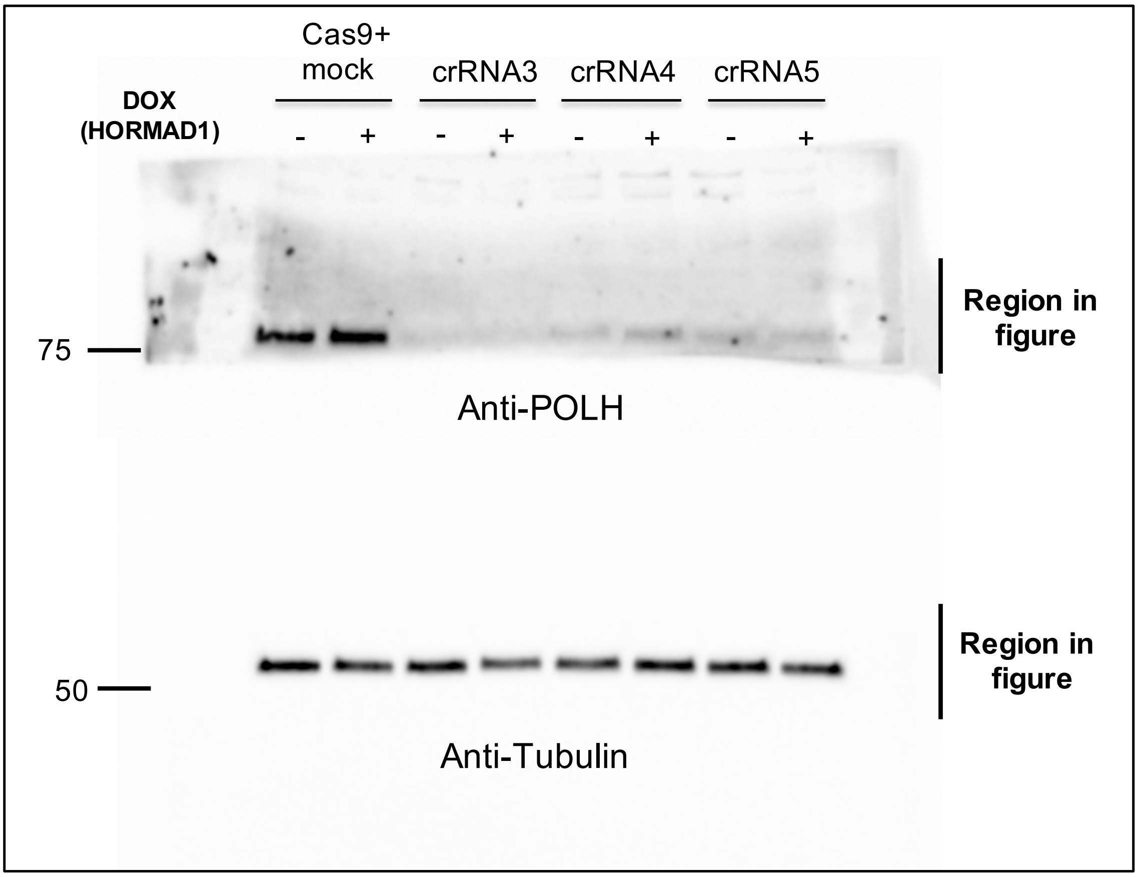
Cas9+ mock
crRNA3
crRNA4
crRNA5
DOX (HORMAD1)
- + - + - + - +
75
Region in figure
Anti-POLH
50
Region in figure
Anti-Tubulin

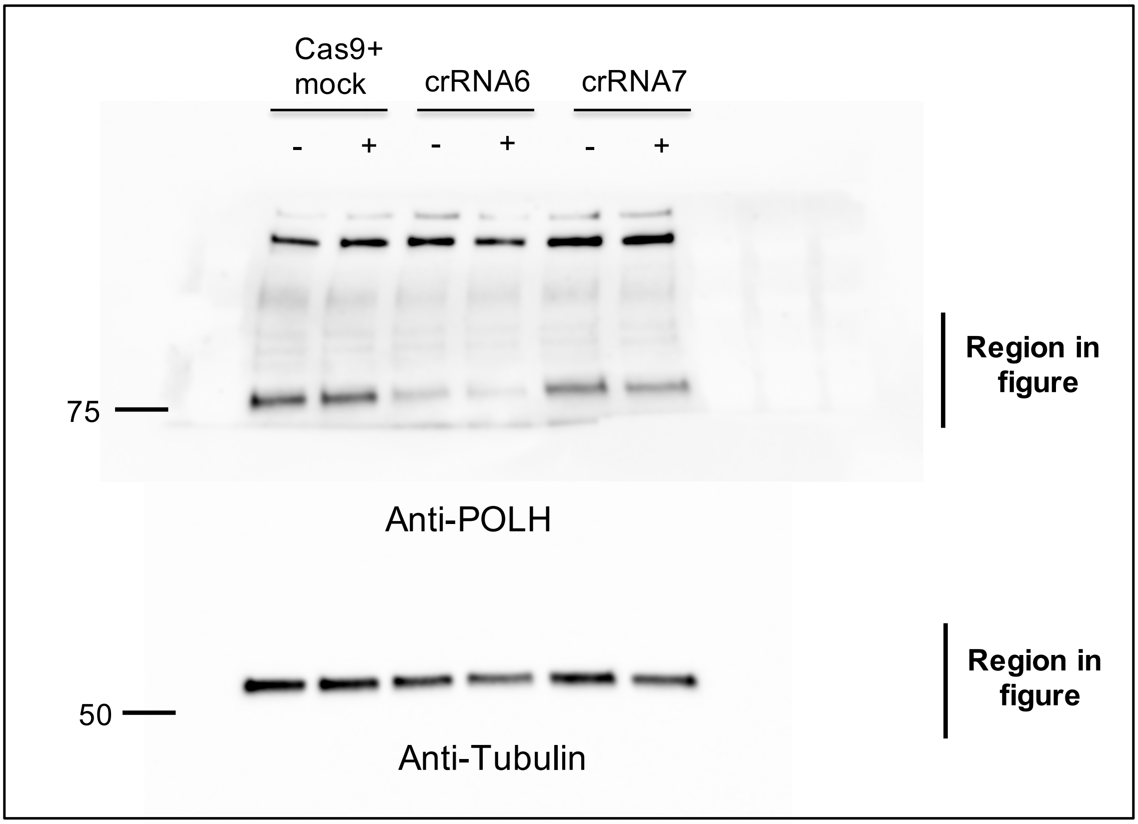
Cas9+ mock
crRNA6
crRNA7
- + - + - +
75
Region in figure
Anti-POLH
50
Region in figure
Anti-Tubulin

Fig. S14

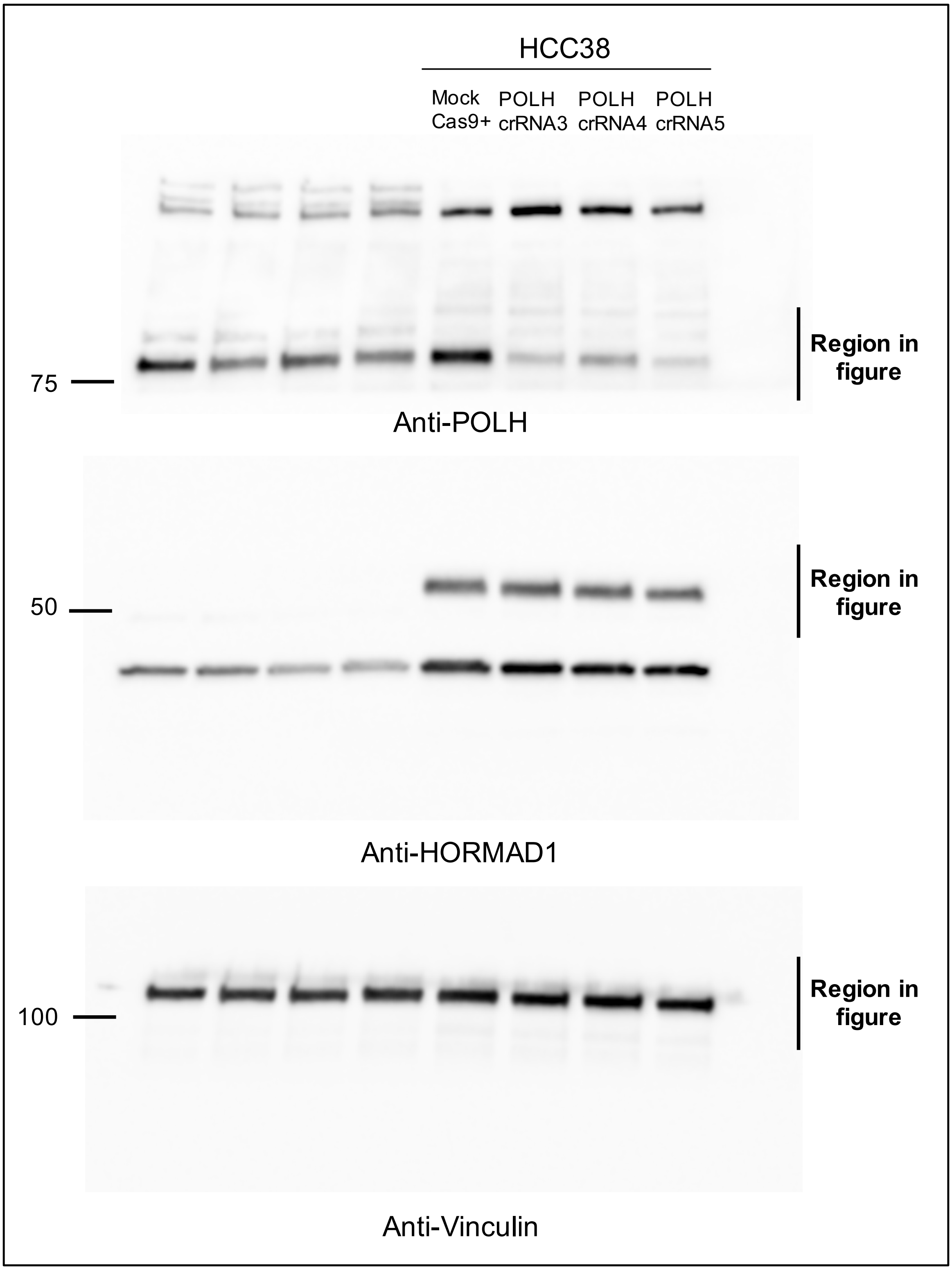
HCC38
Mock Cas9+
POLH crRNA3
POLH crRNA4
POLH crRNA5
75
Region in figure
Anti-POLH
50
Region in figure
Anti-HORMAD1
100
Region in figure
Anti-Vinculin

Fig. S15

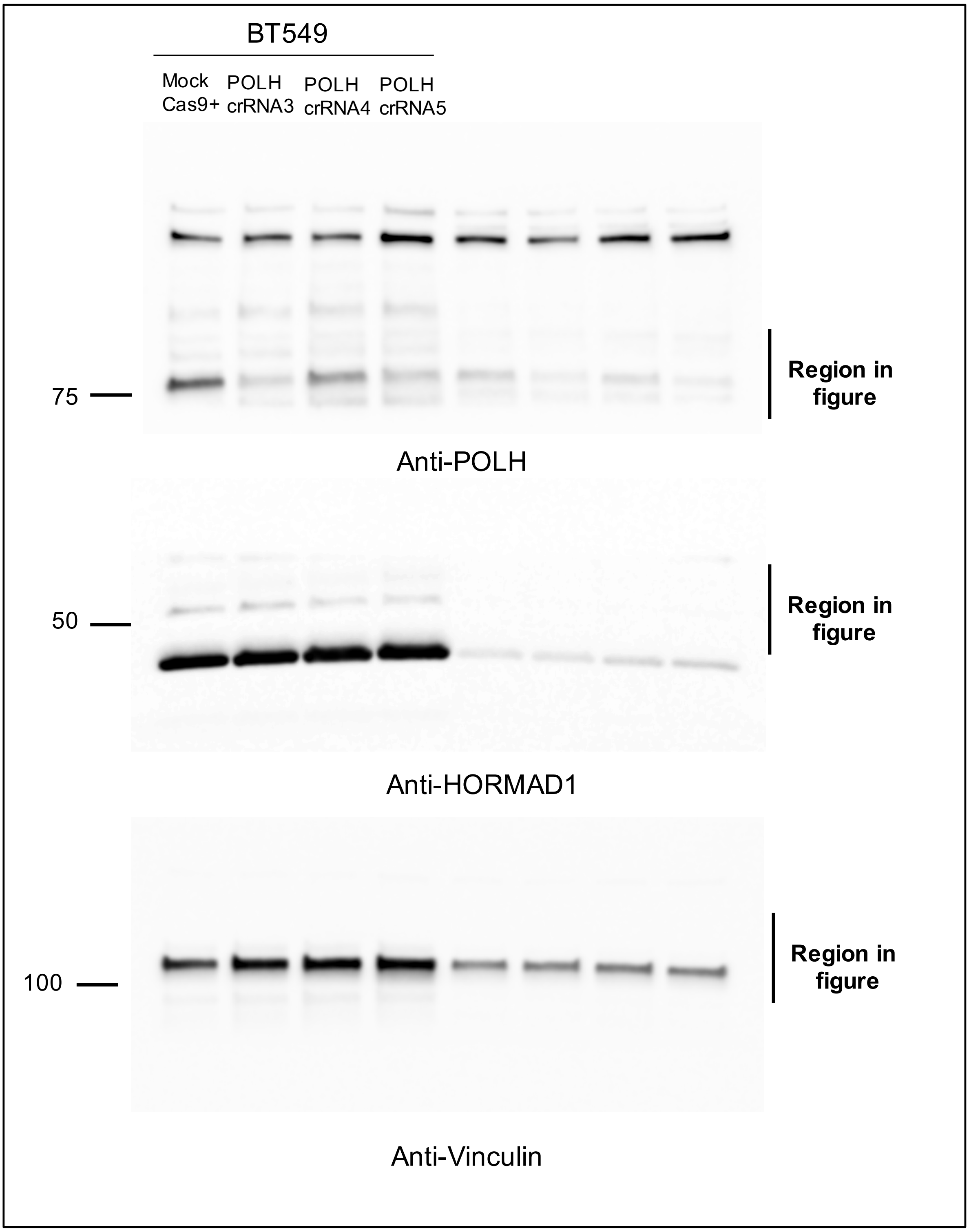
BT549
Mock Cas9+
POLH crRNA3
POLH crRNA4
POLH crRNA5
75
Region in figure
Anti-POLH
50
Region in figure
Anti-HORMAD1
100
Region in figure
Anti-Vinculin

Fig. S16

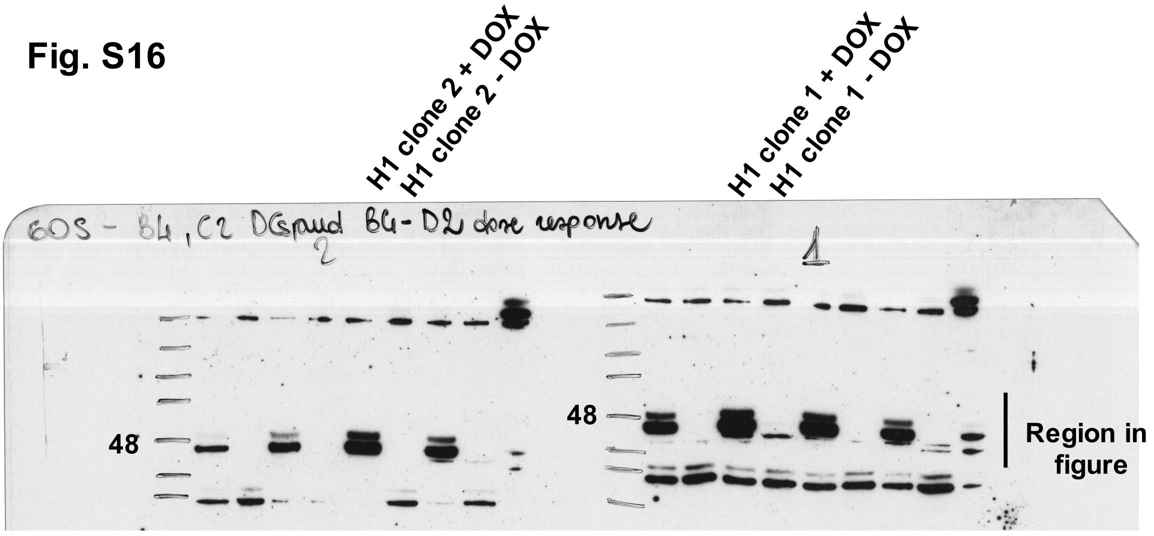

Anti-HORMAD1

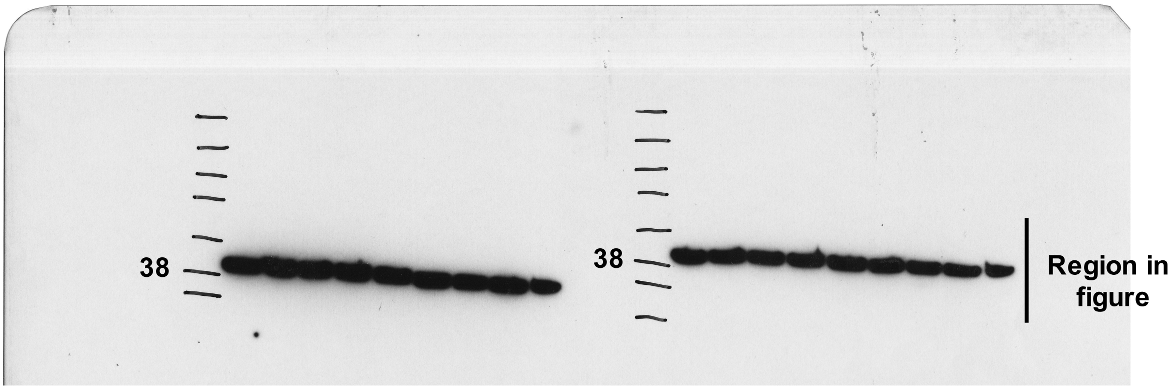

Anti-Actin

Fig. S17

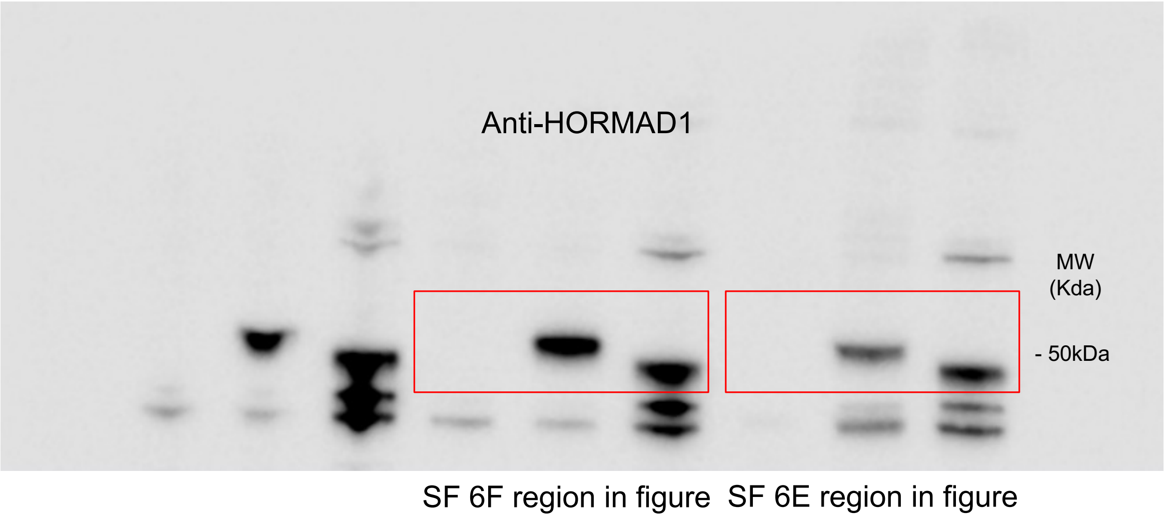

Anti-HORMAD1
MW
(Kda)
- 50kDa
SF 6F region in figure
SF 6E region in figure

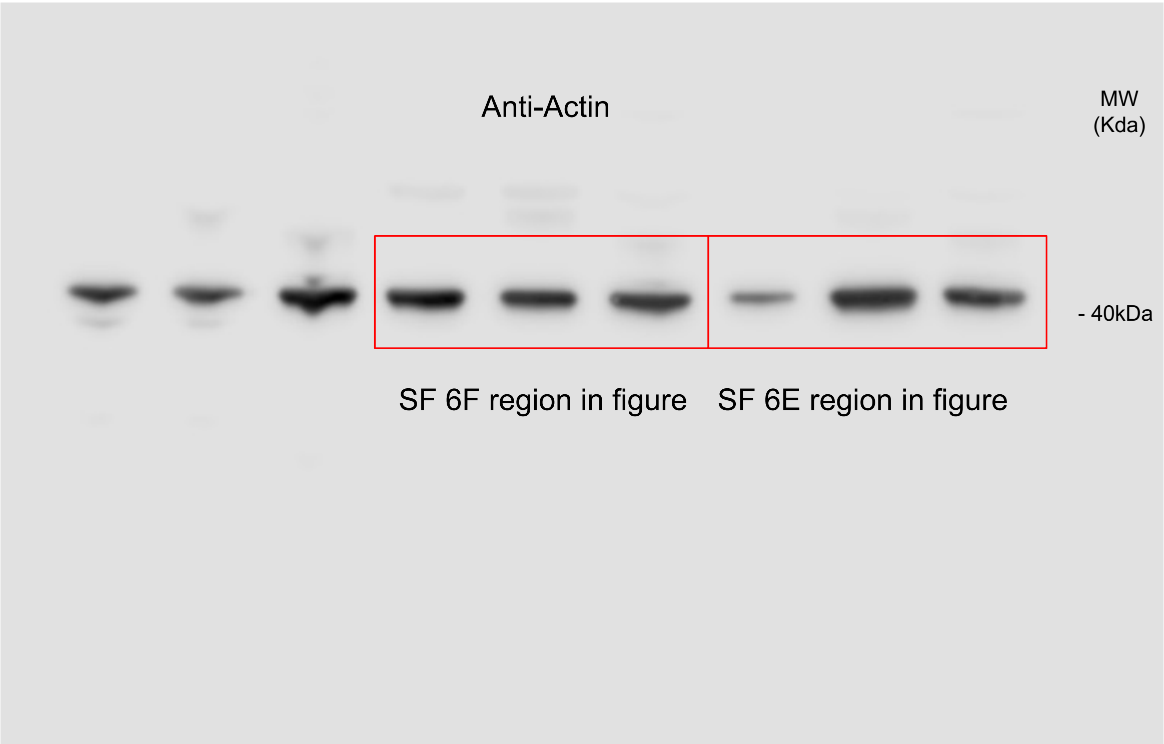

Anti-Actin
MW
(Kda)
- 40kDa
SF 6F region in figure
SF 6E region in figure

Supplementary References

1. Meerbrey KL, Hu GA, Kessler JD, Roarty K, Li MZ, Fang JE, et al. The pINDUCER lentiviral toolkit for inducible RNA interference in vitro and in vivo. P Natl Acad Sci USA. 2011;108(9):3665-70.
2. Jones SE, Fleuren EDG, Frankum J, Konde A, Williamson CT, Krastev DB, et al. ATR Is a Therapeutic Target in Synovial Sarcoma. Cancer Res. 2017;77(24):7014-26.
3. Boutros M, Bras LP, Huber W. Analysis of cell-based RNAi screens. Genome Biol. 2006;7(7):R66.
4. Campbell J, Ryan CJ, Brough R, Bajrami I, Pemberton HN, Chong IY, et al. Large-Scale Profiling of Kinase Dependencies in Cancer Cell Lines. Cell Rep. 2016;14(10):2490-501.
5. Lord CJ, McDonald S, Swift S, Turner NC, Ashworth A. A high-throughput RNA interference screen for DNA repair determinants of PARP inhibitor sensitivity. DNA Repair (Amst). 2008;7(12):2010-9.
6. Patel N, Weekes D, Drosopoulos K, Gazinska P, Noel E, Rashid M, et al. Integrated genomics and functional validation identifies malignant cell specific dependencies in triple negative breast cancer. Nat Commun. 2018;9(1):1044.
7. McComb S, Aguade-Gorgorio J, Harder L, Marovca B, Cario G, Eckert C, et al. Activation of concurrent apoptosis and necroptosis by SMAC mimetics for the treatment of refractory and relapsed ALL. Sci Transl Med. 2016;8(339):339ra70.
8. Concordet JP, Haeussler M. CRISPOR: intuitive guide selection for CRISPR/Cas9 genome editing experiments and screens. Nucleic Acids Res. 2018;46(W1):W242-W5.
9. Szklarczyk D, Gable AL, Lyon D, Junge A, Wyder S, Huerta-Cepas J, et al. STRING v11: protein-protein association networks with increased coverage, supporting functional discovery in genome-wide experimental datasets. Nucleic Acids Res. 2019;47(D1):D607-D13.
